# Taxonomy of *Baetis* Leach in Israel (Ephemeroptera, Baetidae)

**DOI:** 10.3897/zookeys.794.28214

**Published:** 2018-11-01

**Authors:** Zohar Yanai, Jean-Luc Gattolliat, Netta Dorchin

**Affiliations:** 1 School of Zoology, Tel Aviv University, Tel Aviv 6997801, Israel Tel Aviv University Tel Aviv Israel; 2 Musée Cantonal de Zoologie, Palais de Rumine 6, 1014 Lausanne, Switzerland Musée cantonal de zoologie Lausanne Switzerland; 3 Department of Ecology and Evolution, Biophore, University of Lausanne, 1015 Lausanne, Switzerland University of Lausanne Lausanne Switzerland

**Keywords:** Aquatic insects, *
Baetis
*, Israel, mayflies, Palestinian Authority

## Abstract

The taxonomy and systematics of the genus *Baetis* Leach (Ephemeroptera: Baetidae) in Israel is clarified for the first time as part of an ongoing comprehensive survey of the Israeli mayfly fauna. Six clearly defined species are currently recognized in Israel, four of which are described here as new to science. The validity of all species is supported by a molecular analysis of the mitochondrial COI gene. A key for the identification of the Israeli species based on the nymphal stage is provided, as well as data on their distribution patterns and ecology. The local fauna represents four Palearctic species groups, three of which reach their limit of distribution range in Israel. *Baetis* species typically inhabit lotic, pristine habitats in northern Israel, with few exceptions for some species that can be found in the Dead Sea area or in ephemeral ponds.

## Introduction

*Baetis* Leach, 1815 is one of the richest mayfly genera with approximately 160 species worldwide (Barber-James et al. 2008) and more than 80 species in the Palearctic Region (Barber-James et al. 2013). The taxonomy of the genus is subject to ongoing study (Gattolliat and Nieto 2009) and many undescribed and cryptic species are continuously being discovered (e.g., Williams et al. 2006, Leys et al. 2016). For practical reasons, the European species of the genus were divided into eleven species groups by Müller-Liebenau (1969) based on their morphological characters, and this classification is still widely used today. Some of these species groups are considered today as valid genera or subgenera (e.g., Waltz et al. 1994; Thomas and Dia 2000; Jacob 2003).

Some *Baetis* species are widely distributed whereas others are confined to very limited areas. All feed mainly on detritus and periphyton (Bauernfeind and Soldán 2012). In the Levant, nymphs usually occupy habitats of pristine running water, are considered to be sensitive to environmental conditions at least to some extent, and are therefore restricted to more protected habitats. Nevertheless, some species are more tolerant and can be found in more disturbed sites. These characteristics make *Baetis* species ideal candidates for use as bioindicators for water quality (Bauernfeind and Moog 2000, García et al. 2014). However, generating a mayfly-based index necessitates recognition of relevant species and their ecological requirements (Resh and Unzicker 1975), and such knowledge is lacking for the taxonomically difficult Baetidae in many regions. In Israel, the taxonomic knowledge on aquatic insects as a whole is limited (e.g., Botosaneanu and Gasith 1971, Bromley 1988, Wewalka 1989, Dumont 1991), although it is essential for the implementation of bioassessment tools for the local stream systems.

The knowledge about mayflies from the Levant varies with country. The Lebanese and Syrian faunas are relatively well known, mostly thanks to studies by Dia, Thomas and Koch (e.g., Koch 1981, 1988, Thomas and Dia 1983, 1984, 1985, Thomas et al. 1988). By contrast, less than ten species are currently known from Jordan (Gattolliat et al. 2012) and Egypt (Hassan and Abdel Fattah 2007), reflecting the more arid nature of these countries as well as insufficient research.

The Israeli mayfly fauna currently numbers 17 species in seven families (Baetidae, Caenidae, Ephemerellidae, Heptageniidae, Leptophlebiidae, Oligoneuriidae and Prosopistomatidae). Except for Baetidae, the fauna is considered to be well-known, and only limited new discoveries are expected. Samocha (1972) was the first to conduct a thorough investigation on the local mayflies in his MSc thesis, which has never been published. Demoulin (1973) studied the material collected by Samocha and published the first paper on Israeli mayflies, focusing on the Heptageniidae, and Malzacher (1992) and Sartori (1992) provided additional information on most of the remaining families. By contrast, mayflies have almost never been studied in the Palestinian Authority; Samocha (1972) sampled a handful of sites that yielded few records, followed by sporadic field surveys by Israeli ecologists, all suggesting that the fauna in this region is similar, yet poorer, than that of semi-arid regions in Israel.

The Baetidae, by far the richest and most common family in Israel, remain poorly studied. Samocha (1972) reported 20 morphospecies from the country (although the true number must be lower because he did not associate nymphs and adults), and included only one named species, “*Cloeondipterum*” (Linnaeus, 1761). *Baetissamochai* Koch, 1981 and *B.monnerati* Gattolliat & Sartori, 2012 were subsequently described from Lebanon, Syria, or Jordan and are reported here from Israel for the first time.

In the present paper, we provide the first comprehensive taxonomic treatment of the genus *Baetis* in Israel, based on integrative study of nymphal morphology and molecular analysis. We include a key to nymphs of all species and a description of four new species. The present study contributes to a better understanding of the Israeli mayfly fauna and may promote the development of local biological indices for water quality.

## Materials and methods

**Figure 1.**
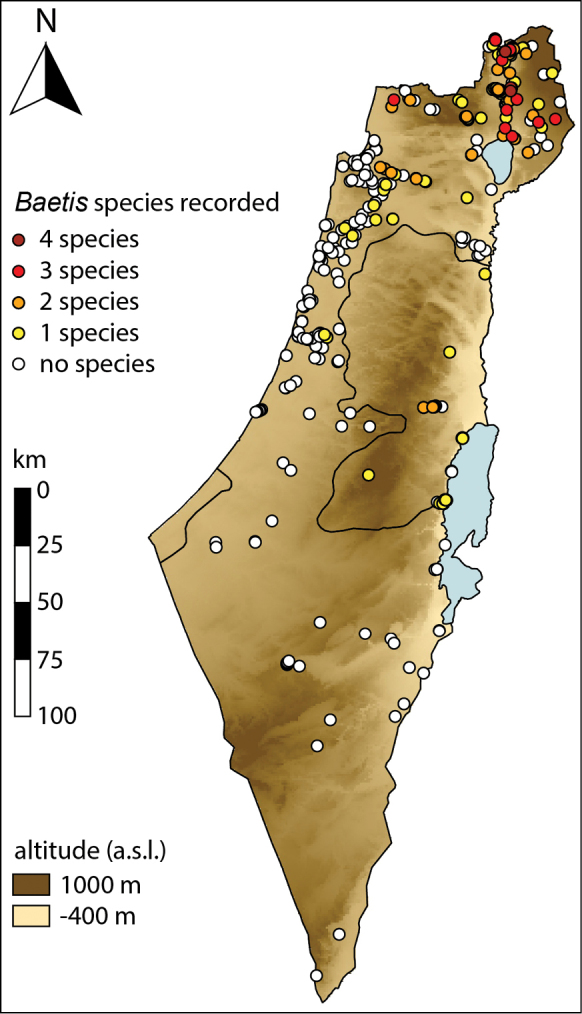
Sampling sites and *Baetis* species richness in Israel and the Palestinian Authority.

**Figure 2.**
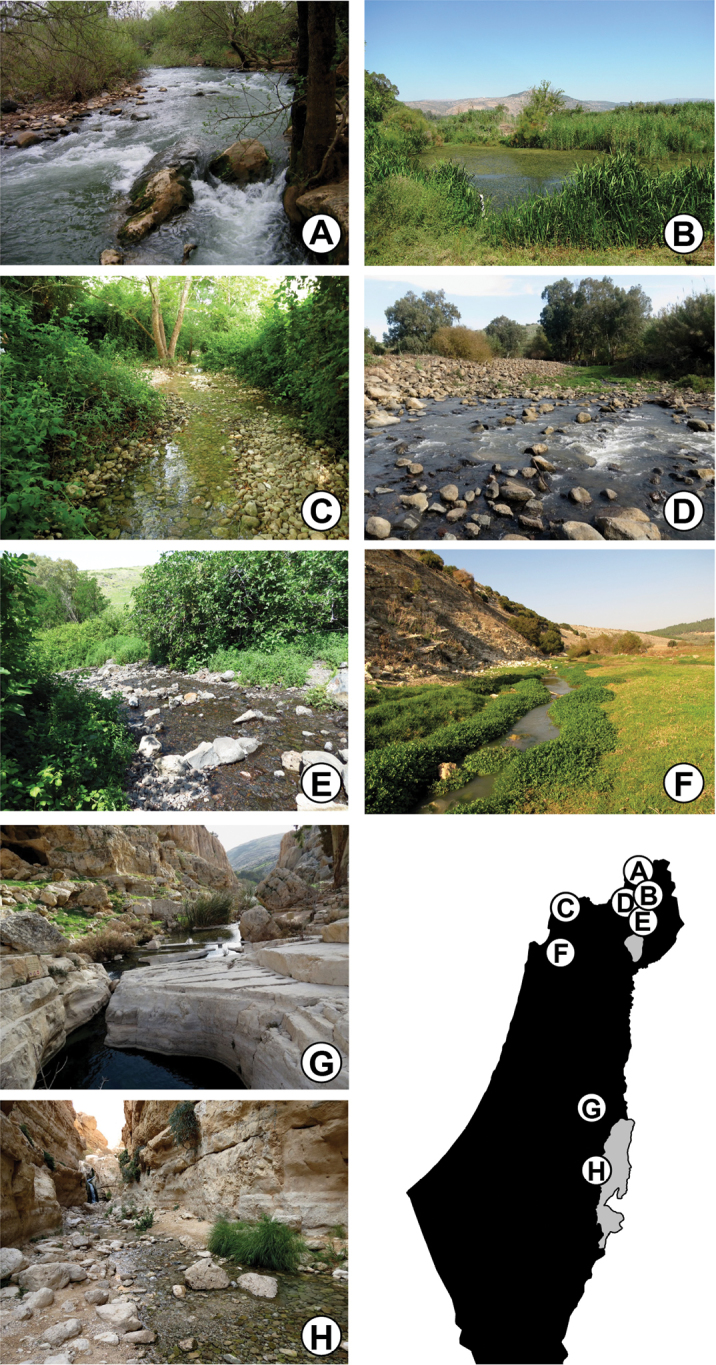
Typical *Baetis* habitats in Israel and the Palestinian Authority: **A** Senir Stream, a main source of the Jordan River **B** Hula nature reserve, a marshland habitat **C** Keziv Stream, a pristine stream in a forested area in the Upper Galilee **D** Ateret Fortress, a site in the Upper Jordan River system **E** Maymon Spring, a small spring-fed brook in the Upper Jordan River system, the Golan Heights **F** Zippori Stream, a lowland stream in the Lower Galilee **G** Perat Stream (Wadi Qelt) in the eastern Judean Mountains **H** Arugot Stream, a desert stream in the arid water system of the Dead Sea.

Collecting was done in over 400 sites around Israel and the Palestinian Authority (Figure 1; Supplementary File 1) between 2014–2018 throughout the year, and covered diverse types of aquatic habitats (Figure 2). *Baetis* nymphs were collected using a hand net or picked manually from rocks and pebbles and preserved in ethanol. Collected specimens were studied under a Leica M125 or M205 stereomicroscope. Microscope slides were prepared from body parts important for diagnosis, fixed in Canada Balsam, and drawn from a drawing tube mounted on a Leica DM1000 or Olympus BX51 compound microscopes. Morphological descriptions followed Hubbard (1995). Body surface of *Baetis* nymphs is usually covered by scales and setae on enlarged bases. These tend to be lost often during the mounting process, and therefore do not appear in most microscope slides and resulting figures (Kluge et al. 2018). Measurements in the following descriptions are based on 20–36 individuals for each species. The material examined included nymphs only. A small number of adults was collected in the field or reared in the lab, but not enough information is available to associate them with the nymphal stages, therefore they will be dealt with in a future study. Most of the material used in this study, whether preserved in ethanol or on microscope slides, is deposited in the Steinhardt Museum of Natural History at Tel Aviv University, Israel (SMNHTAU), including all holotypes and most paratypes. Remaining paratypes are deposited in the Musée Cantonal de Zoologie at Lausanne, Switzerland. Museum personal identifiers are mentioned for holotypes.

For the phylogenetic reconstruction we used freshly collected nymphs from populations throughout the distribution range of each species in Israel. DNA was extracted using the DNAeasy blood and tissue kit and BioSprint 96 extraction robot (Qiagen Inc., Hilden, Germany), implementing the non-destructive protocol outlined by Vuataz et al. (2011), which enabled post-extraction morphological study of specimens. The ‘barcoding section’ of the mitochondrial cytochrome *c* oxidase subunit I (COI) was PCR-amplified with the primers HCO2198 and LCO1490 (Folmer et al. 1994). Polymerase Chain Reaction was conducted in a volume of 25–30µl, consisting of 2–6µl DNA template, 1µl of 10 pmol/µl of each primer, 2.5 µl 10 mM dNTP solution, 3–3.5 20 mM MgSO4, 2.5µl Taq buffer, and 1 unit Taq polymerase. Optimized PCR conditions included initial denaturation at 94°C for 5min, 35–40 cycles of denaturation at 94°C for 30–40 s, annealing at 47–54°C for 30–45 s, and extension at 72°C for 40–60 s, with final extension at 72°C for 7–10min. Automated sequencing was carried out in Hy Laboratories (Rehovot, Israel) or in Microsynth (Balgach, Switzerland).

Molecular reconstruction was conducted on 48 newly obtained sequences, as well as three *B.monnerati* specimens from Jordan (Gattolliat et al. 2012), and four reference sequences obtained from GenBank (http://www.ncbi.nlm.nih.gov/Genbank) and from Rutschmann et al. (in prep.), to represent the four species groups dealt with in this study. All GenBank accession numbers and collecting details of the specimens are given in Supplementary File 2. Sequence chromatograms were inspected and edited using Geneious v.7.1.5 (Biomatters Ltd.). Alignment, reconstruction, and genetic distance calculations were conducted in MEGA v.7.0 (Kumar et al. 2015). We selected GTR+G+I with partial deletion as the most appropriate model for reconstruction (based on lowest AICc and BIC scores), and conducted a Maximum Likelihood analysis with 1000 bootstrap replicates.

## Results and discussion

**Figure 3.**
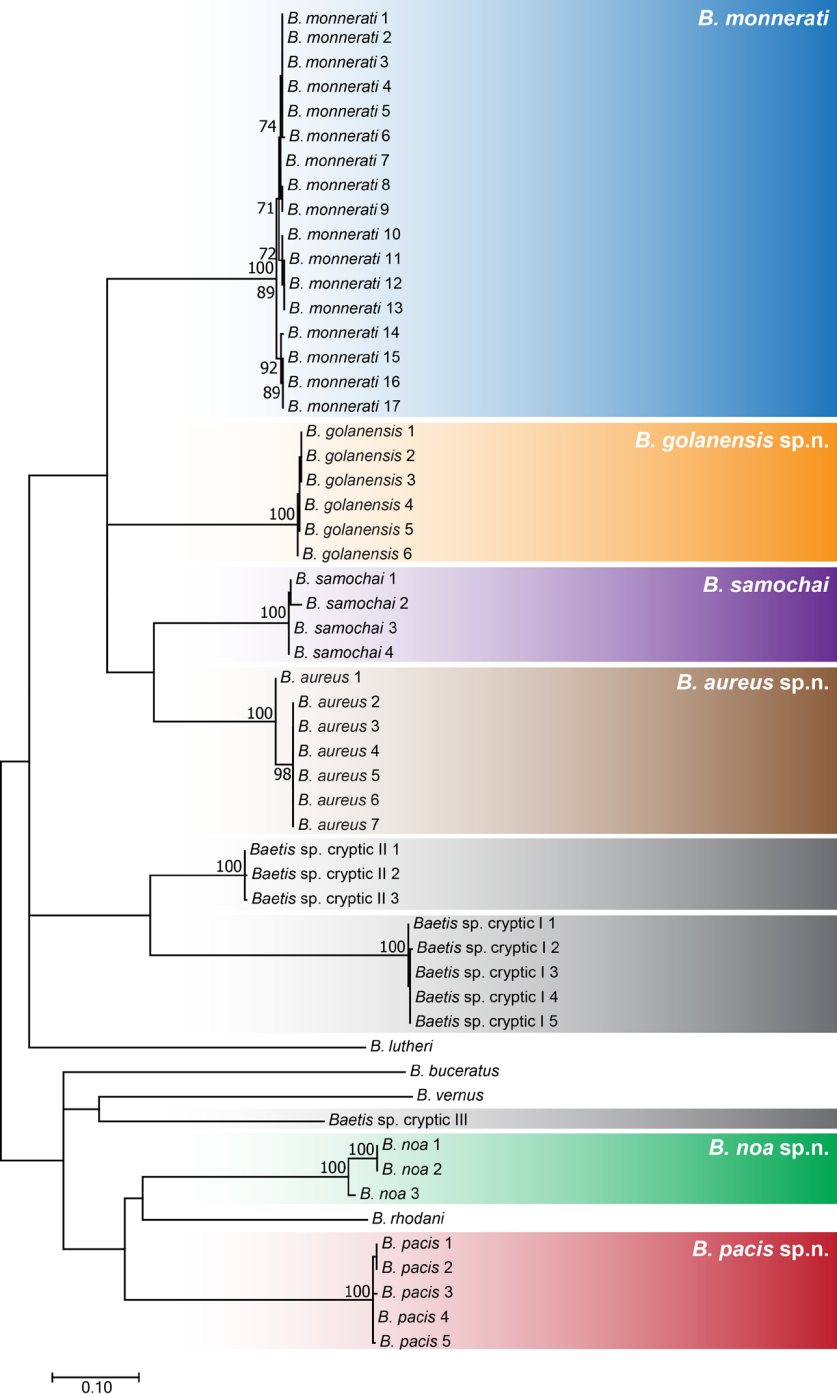
A Maximum Likelihood phylogenetic reconstruction based on sequences of the mitochondrial COI gene obtained from Israeli and related species. The validity of six Israeli species is confirmed and strongly supported. Gray shaded clades represent cryptic species that are morphologically indistinguishable from the species described here. Bootstrap values above 70% are indicated next to the nodes.

In the present study, two described and four new species of *Baetis* were found in Israel, all are well supported by the molecular phylogenetic analysis of mitochondrial COI sequences (Figure 3). Inter- and intraspecific genetic differences were found to be ≥11.9% and ≤3.5%, respectively (Table 1). The phylogenetic analysis also identified three independent and highly supported clusters, representing groups of individuals which could not be distinguished morphologically and did not differ in their distribution patterns from the six recognized species (cryptic species I with *Baetisgolanensis*, cryptic species II with *B.monnerati*, and cryptic species III with *B.noa*). We consider them to be cryptic species but refrain from describing them until further morphological, molecular, and ecological studies.

**Table 1.** Intraspecific (bold) and interspecific genetic distances among sequences of the mitochondrial COI gene of the six Israeli *Baetis* species (mean, minimum–maximum).

	* B. samochai *	* B. pacis *	* B. noa *	* B. monnerati *	* B. aureus *	* B. golanensis *
* B. samochai *	**0.5**%					
	**0.0%–0.7**%					
* B. pacis *	26.5%	**0.3**%				
	26.1%–27.1%	**0.0%–0.7**%				
* B. noa *	18.7%	22.0%	**2.3**%			
	17.7%–19.4%	21.3%–22.3%	**0.0%–3.5**%			
* B. monnerati *	13.5%	18.6%	20.6%	**0.7**%		
	11.9%–15.3%	17.7%–19.6%	18.6%–21.3%	**0.0%–2.1**%		
* B. aureus *	12.4%	23.0%	18.6%	13.9%	**0.0**%	
	12.0%–12.8%	22.2%–23.1%	18.6%–18.6%	13.6%–14.5%	**0.0%–0.0**%	
* B. golanensis *	13.5%	27.0%	15.5%	15.0%	14.3%	**0.0**%
	13.5%–13.5%	27.0%–27.1%	15.2%–16.0%	14.3%–15.2%	14.3%–14.3%	**0.0%–0.0**%

### Treatment of species

#### Genus Baetis

Taxon classificationAnimaliaEphemeropteraBaetidae

Leach, 1815

##### Diagnosis.

The main diagnostic morphological characteristics of the genus pertain to nymphs and include the cylindrical body, two pairs of wing pads, absence of setae between prostheca and mola of both mandibles, simple stout prostheca on both mandibles, presence of femoral villopore, robust tarsal claws with a single row of denticles, gills consisting of a single lamella on abdominal segments I–VII, unmodified paraproct and three caudal filaments (Bauernfeind and Soldán 2012). The present study is focused only on *Baetis* s.s. and the following descriptions include only nymphs.

#### Baetis aureus

Taxon classificationAnimaliaEphemeropteraBaetidae

Yanai & Gattolliat sp. n.

http://zoobank.org/C06B5ED9-2EA6-4DAE-AD1A-73EF8112F0C7

Figures 4A , 5 , 6

##### Differential diagnosis.

*Baetisaureus* is unique within its potential species groups in having a relatively short median caudal filament (half-length of cerci) and scale bases on the surface of the abdominal terga only slightly wider than seta bases. In Israel it is distinguishable by the long gills, elongate body, and wide, triangular spines along the distal margin of terga.

##### Description.

*Length* (mature nymphs). Female (n = 17): 4.2–4.5 mm (summer generation) to 5.3–7.2 mm (winter generation); cerci 2.6–5.4 mm; median caudal filament 1.7–2.7 mm. Male (n = 8): 4.5–4.7 mm (summer) to 6.0–7.1 mm (winter); cerci 2.9–4.1 mm; median caudal filament 1.6–2.6 mm.

**Figure 4.**
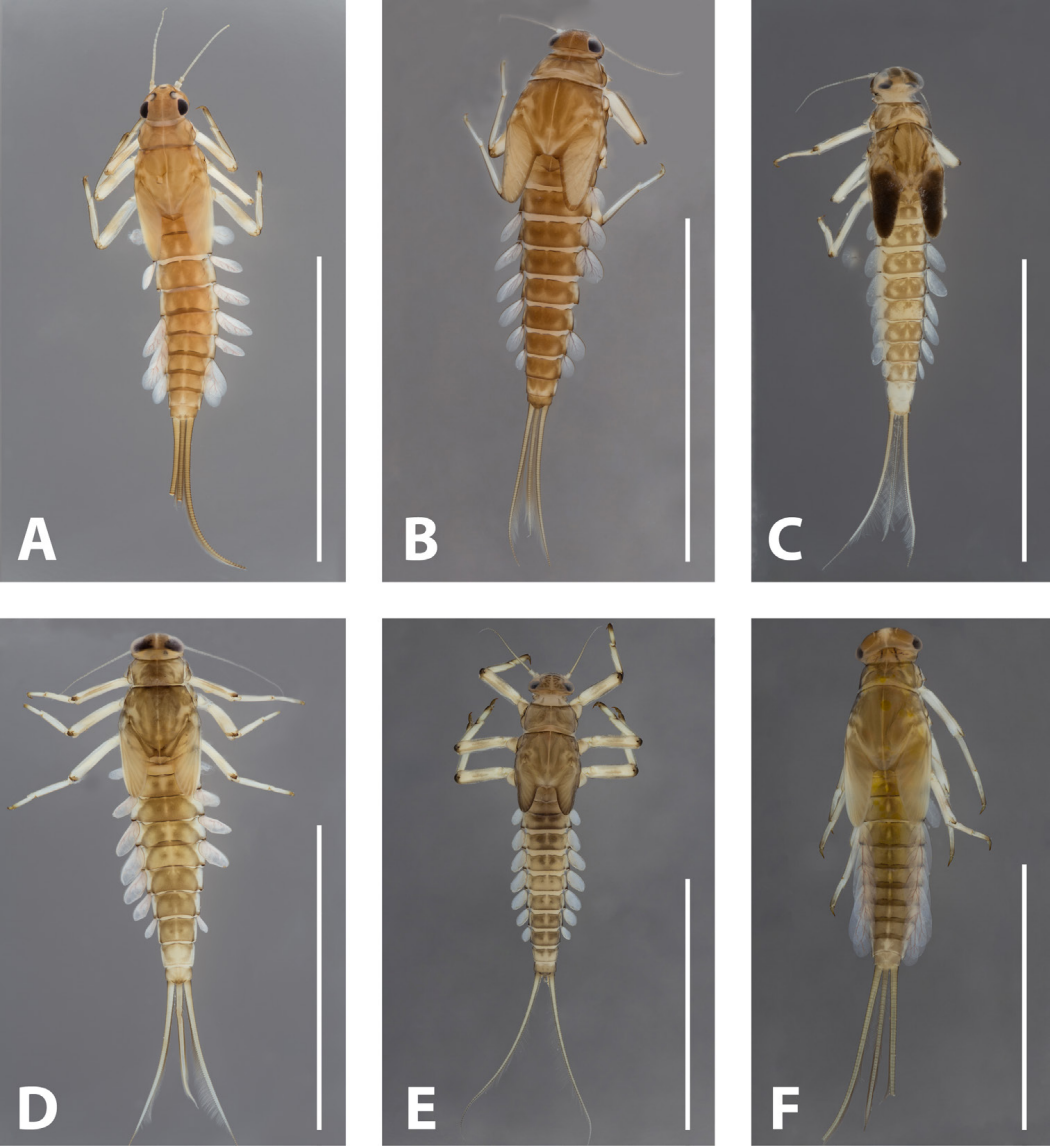
Nymphs of Israeli *Baetis* species, habitus. **A***Baetisaureus* sp. n. **B***Baetisgolanensis* sp. n. **C***Baetismonnerati***D***Baetisnoa* sp. n. **E***Baetispacis* sp. n. **F***Baetissamochai*. Scale bars: 5 mm.

*Colouration.* General colour light brown to yellow (Figure 4A). Head light brown, antennae, clypeus and labrum ecru. Turbinate eyes in male nymph amber-orange. Thorax brown with a few pale marks but without clear pattern. All legs whitish, brownish hue sometimes on middle of trochanter and femur, brown shade on claw and distal 1/3 of tarsus. Abdominal terga yellowish brown, with pale narrow posteriomedial triangular spot forming dashed dorsal line along abdomen. Abdominal sterna ecru to pale yellow. Gills milky, semi-transparent, costal margins darker, tracheation brownish purple. Cerci and median caudal filament uniformly ecru without bands or pattern.

**Figure 5.**
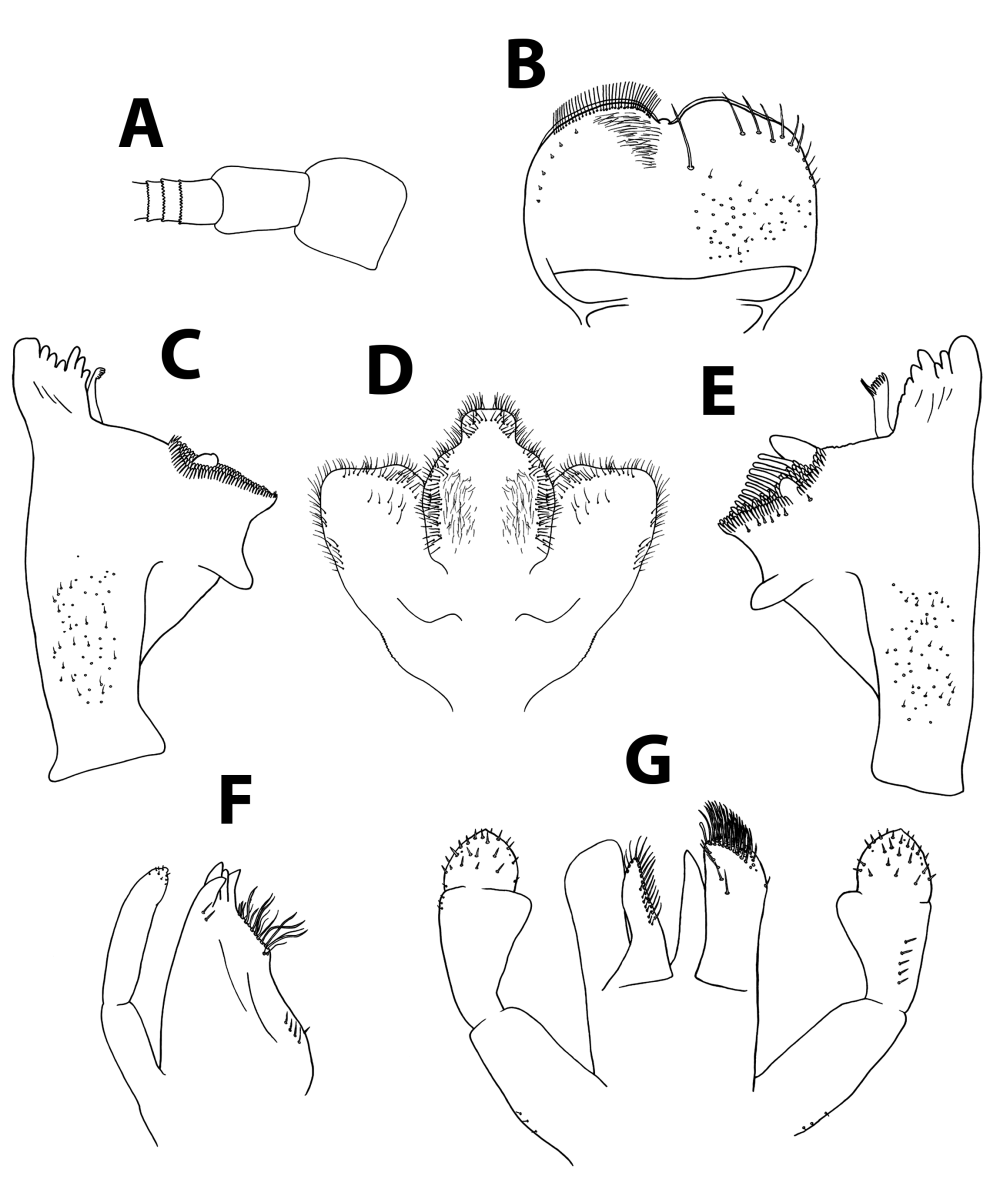
*Baetisaureus* sp. n., nymph. **A** antennal scape, pedicel and first flagellomeres **B** labrum (left, ventral; right, dorsal) **C** right mandible **D** hypopharynx **E** left mandible **F** maxilla **G** labium.

*Head.* No carina between antennae; pedicel almost bare (Figure 5A). Labrum (Figure 5B): dorsal surface with scattered, fine, short setae and seta bases, with one median pair of long setae; lateral margins each with distal arc of 5–6 long, simple, stout setae and 4–5 lateral setae; ventral surface with 5–6 small, stout setae laterodistally; distal margin with row of 30–40 fine, long, feathered setae. Hypopharynx (Figure 5D): lingua tri-lobed; surface covered with minute hair-like setae, denser and stouter apically; lingua and superlingua covered with minute, stout setae; base of superlingua laterally serrated. Right mandible (Figure 5C) with incisors composed of two sets of four and three denticles, outer denticles more prominent than others; prostheca with 6–7 denticles; mola apex with tuft of setae. Left mandible (Figure 5E) with incisors composed of six denticles, outer one broadest and most prominent; prostheca with six denticles and few long, soft setae; space between prostheca and mola slightly crenulate; mola apex without tuft of setae; base of mola with few spines. Maxillae (Figure 5F) with four broad teeth; lacinia with one row of small setae and long, serrated setae; row of 4–6 setae at base of lacinia; one seta perpendicular to lacinia margin; palpus 2-segmented, segments of similar length; segment II with small nipple, covered with sparse minute setae, apical scale absent. Labium (Figure 5G): glossa slightly shorter than paraglossa; glossa inner margin and apex with long, simple setae; paraglossa not curved; apex with apical stout, blunt seta and distal quarter with three rows of long setae; labial palpus 3-segmented; segment I as long as segments II and III combined; segment II with noticeable distolateral protuberance (“thumb”) and dorsal row of 5–7 long, pointed setae.

**Figure 6.**
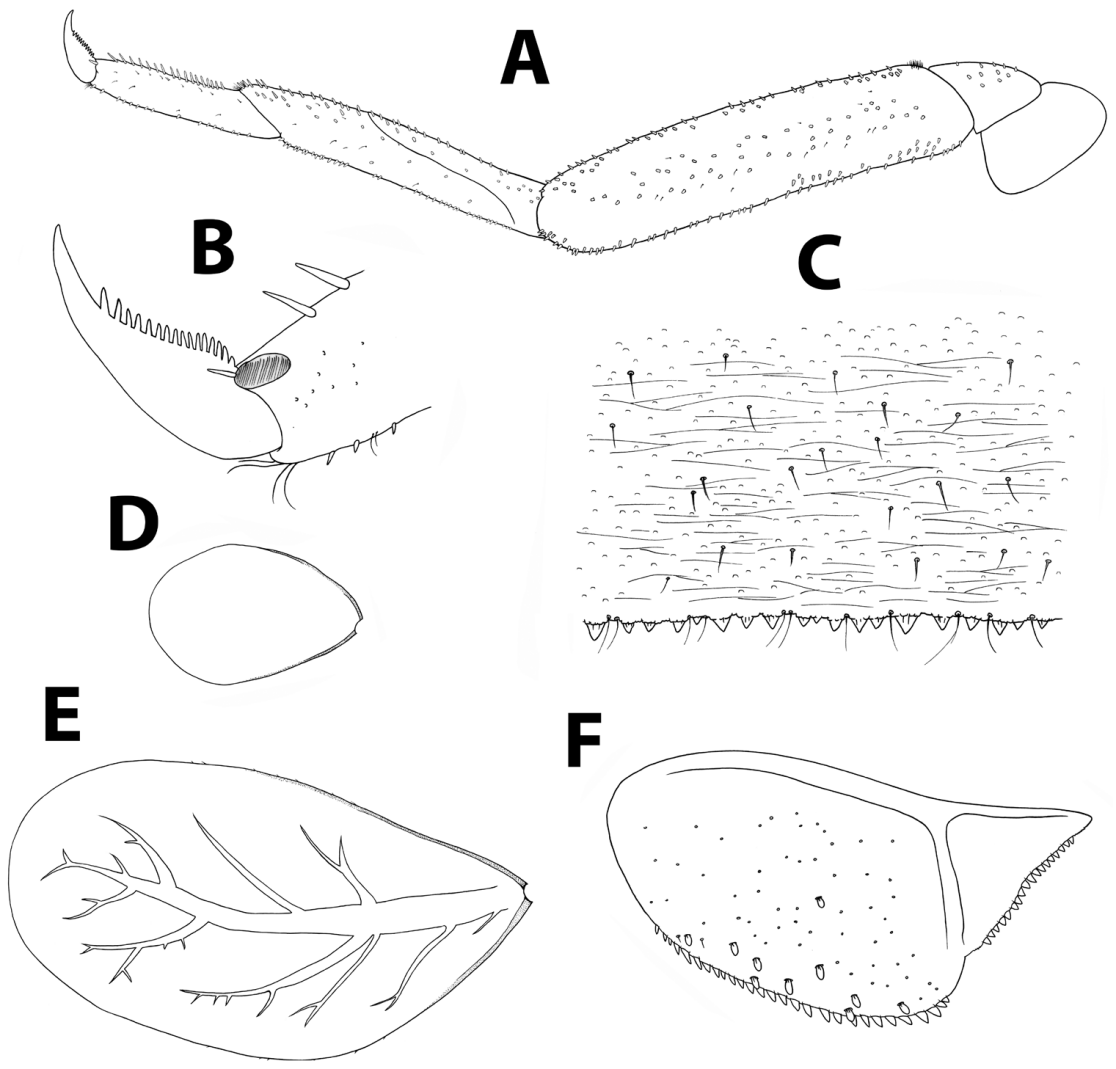
*Baetisaureus* sp. n., nymph. **A** foreleg **B** tarsal claw **C** abdominal tergum V, surface and distal margin **D** gill I **E** gill IV **F** paraproct plate.

*Thorax.* Forelegs (Figure 6A): coxa almost bare. Trochanter with few short, stout setae. Femora with dorsal row of ca. 50 short, stout setae, and ventral row of ca. 30 minute setae, both evenly distributed along margin; dorsoapical setal patch composed of 5–7 stout setae and 2–3 thin setae. Tibiae with dorsal and ventral rows of stout, pointed setae; tibiopatellar suture present. Surface of trochanters, femora and tibiae with scattered feathered scales. Tarsi with 10–20 minute dorsal setae; 13–20 ventral pointed setae and submarginal row of shorter setae; one pointed seta, shorter than most ventral setae, at ventral tarsus-claw meeting point. Tarsal claws (Figure 6B) hooked, with row of 15–17 acute teeth. Mid- and hindlegs similar to forelegs.

*Abdomen.* Terga shagreened with few thin setae, seta bases and very small scale bases (only slightly wider than seta bases); distal margin of all terga with row of triangular spines as broad as long (spines longer and more pointed on terga VII to IX), with spaces between them no broader than spine length (Figure 6C). Gill I oval, reduced, without marginal spines and visible tracheation (Figure 6D); gills II to VII elongate, 1.7 times longer than following tergum; margins thicker with very fine serration in proximal half; branched tracheae clearly visible (Figure 6E). Paraproct (Figure 6F) with elongate scales, hair-like setae and seta bases; margin with 20–30 regular triangular spines; postero-lateral extension with few scales, margin with 14–20 triangular spines of similar size.

##### Affinities.

*Baetisaureus* exhibits the main characters of the *B.vernus* and *B.buceratus* species groups sensu Müller-Liebenau (1969) and cannot be assigned to any of them with certainty. Therefore, it is compared here to species of both species groups. Characters typical to both groups include mandibles with bigger outer tooth, labrum with submarginal arc of less than eight setae, tarsal claws without subapical setae, distal margin of abdominal terga with triangular spines, relatively long gills, and generally inconspicuous pattern of the terga (Bauernfeind and Soldán 2012). *Baetisaureus* differs from all known species of these two species groups by the median caudal filament that is slightly longer than half the length of its cerci (whereas in other species the median caudal filament is almost as long as the cerci, except in *B.zdenkae* Soldán & Godunko, 2009), and by the small size of the scale bases on the surface of the abdominal terga.

In the *B.vernus* species group, seven species were reported from the West Palearctic region, and differ from *B.aureus* as follows: *Baetisvernus* Curtis, 1834 has a mola with two small auxiliary spines at the outer side of the subtriangular process (figure 91 in Eiseler 2005). In *B.liebenauae* Keffermüller, 1974 the outer sets of incisors in the mandibles are fused, and the spines on the distal margin of terga are blunt and rounded (Keffermüller 1974). In *B.macrospinosus* Koch, 1985 the setae on the dorsal margin of femora are longer and apically rounded, terga are covered with abundant scale bases, spines on distal margin of terga are blunt and rounded, and dorsal pattern of abdomen is well-marked (Koch 1985). In *B.macani* Kimmins, 1957 the distolateral protuberance on segment II of the labial palpus (“thumb”) is considerably developed (Müller-Liebenau 1969). The gill tracheation of *B.tracheatus* Keffemüller & Machel, 1967 is conspicuous. The submarginal arc of setae on the labrum of *B.tracheatus* and *B.subalpinus* Bengtsson, 1917 is reduced to 2–3 setae (Müller-Liebenau 1969). Finally, *B.samochai* Koch, 1981 is characterized by long and sharp spines along the distal margin of terga, its maxillary palpus is longer than the galealacinia, and the dorsal pattern on its abdomen is different (Koch 1981).

*Baetisaureus* differs from all other known species of the *B.buceratus* species group (see Soldán and Godunko 2009) as follows: in *B.buceratus* Eaton, 1870, the submarginal distal arc on the labrum consists of only 3 setae, and spines on the distal margin of terga are narrow and blunt (Müller-Liebenau 1969). In *B.spei* Thomas & Dia, 1985, a geographically close relative from Lebanon, the shape of the labial palpus is different, teeth on the outer set of the left mandible incisor are wider, spines on the distal margin of terga are rounded and blunt, marginal spines on paraproct are irregular and of variable shapes (Thomas and Dia 1985). In *B.pentaphlebodes* Ujhelyi, 1966 segment III of the labial palpus is shorter (compared to segment II). In *B.zdenkae* from Rhodes, Greece, the incisors of the right mandible are not arranged in a decreasing size order, more setae are present on the paraglossa base, and less marginal spines are found on the paraproct (Soldán and Godunko 2009). Finally, in *B.monnerati* the setae on the dorsal margin of femora are long, and the gills are oval (Gattolliat et al. 2012).

##### Etymology.

*Aureus* (Latin for golden) refers to the general colour of the nymph body.

##### Distribution and ecology.

This species is common in the northern regions of Israel: Lower and Upper Galilee, Hula Valley and Golan Heights, and rare in the Yarqon stream. It is found in habitats of running, pristine water, particularly in small creeks but also in larger streams such as the Keziv and Zippori (Figure 2C, E, F). The substrate is usually composed of stones of different sizes, and localities with dense submerged vegetation are preferred. Mature nymphs were collected mostly in spring (March–May) and fall (October–November).

##### Material examined.

**Holotype**. ISRAEL: 1 Nymph, Gamla Stream (Peham Springs), 32.9672°N, 35.8201°E, ca 690 m a.s.l., 04.iv.2016, Z. Yanai, SMNHTAU291999. **Paratypes**. ISRAEL: 88N, Barqan Stream, 25.v.2011, Y. Hershkovitz; 1N, Gilbon Stream (upstream Devora Waterfall), 09.iv.2014, Z. Yanai; 1N, Yarqon Stream (national park), 10.iv.2014, Z. Yanai; 4N (1N on slide), Ammud Stream (Yaqim Spring), 20.v.2014, Z. Yanai; 1N, Fit Spring, 10.vi.2014, Z. Yanai; 9N (2N on slides), Gaaton Junction, 17.vi.2014, Z. Yanai & D. Mayer; 3N, Zippori Stream (Ras Ali), 02.iii.2015, Z. Yanai; 1N, Gilbon Stream (upstream Devora Waterfall), 29.x.2015, Z. Yanai & Y. Brenner; 107N (1N on slide), Keziv Stream (Hardalit Spring), 07.xi.2015, Z. Yanai & S. Cohen; 17N, Keziv Stream (Tamir Spring), 07.xi.2015, Z. Yanai & S. Cohen; 1N, Zippori Stream (Zippori Springs), 07.xi.2015, Z. Yanai & S. Cohen; 40N, Zippori Stream (Ras Ali), 07.xi.2015, Z. Yanai & S. Cohen; 19N, Gamla Stream (Peham Springs), 04.iv.2016, Z. Yanai; 154N (1N on slide), Zippori Stream (Zippori Springs), 19.iv.2016, Z. Yanai; 24N (1N on slide), Rosh Pinna Stream (Rosh Pinna), 15.x.2016, Z. Yanai; 6N, Yarqon Stream, 14.v.2017, Y. Hershkovitz. **Other material**. ISRAEL: 4N, Zippori Stream (Yivqa Spring), 21.ii.2014, Z. Yanai; 5N (1N on slide), Keziv Stream (Hardalit Spring), 17.vi.2014, Z. Yanai & D. Mayer; 4N, Ammud Stream (Poem Spring), 18.v.2015, Y. Hershkovitz & T. Eshcoly.

#### Baetis golanensis

Taxon classificationAnimaliaEphemeropteraBaetidae

Yanai & Gattolliat sp. n.

http://zoobank.org/08E663AD-EAF1-4DE4-B70C-CA52AA808552

Figures 4B , 7 , 8

Baetis A12: Samocha 1972: 44–45, pl XVIII, figs 7–9; pl XIX, figs 1–9.
Baetis lutheri : Koch 1988: 93.

##### Differential diagnosis.

Among the species with well-developed median caudal filaments in the *B.lutheri* species group, *B.golanensis* is the only one with a combination of the following characters: right mandibular incisors of similar size, irregular blunt quadrangular spines on the distal margin of terga, and non-sclerotized protuberances next to the coxae. This is the only species in Israel with a developed median caudal filament that also has ventral protuberances near coxa bases and subapical setae on the claws.

##### Description.

*Length* (mature nymphs). Female (n = 11): 5.1–6.3 mm; cerci 2.2–2.9 mm; median caudal filament 1.4–2.4 mm. Male (n = 9): 4.6–6.1 mm; cerci 2.2–3.0 mm; median caudal filament 1.7–2.4 mm.

*Colouration.* General colour brown (Figure 4B). Head brown, antennae ecru, area around compound eyes and ocelli ecru. Turbinate eyes in male nymph amber. Thorax dark brown. Legs ivory to ecru, claws, apex of femora and tibiae brown. Abdominal terga uniformly brown, terga V, IX and X sometimes distally lighter. Abdominal sterna ivory to beige. Gills almost transparent, tracheation light brown. Cerci ecru to brown, without bands or pattern, median caudal filament sometimes lighter.

**Figure 7.**
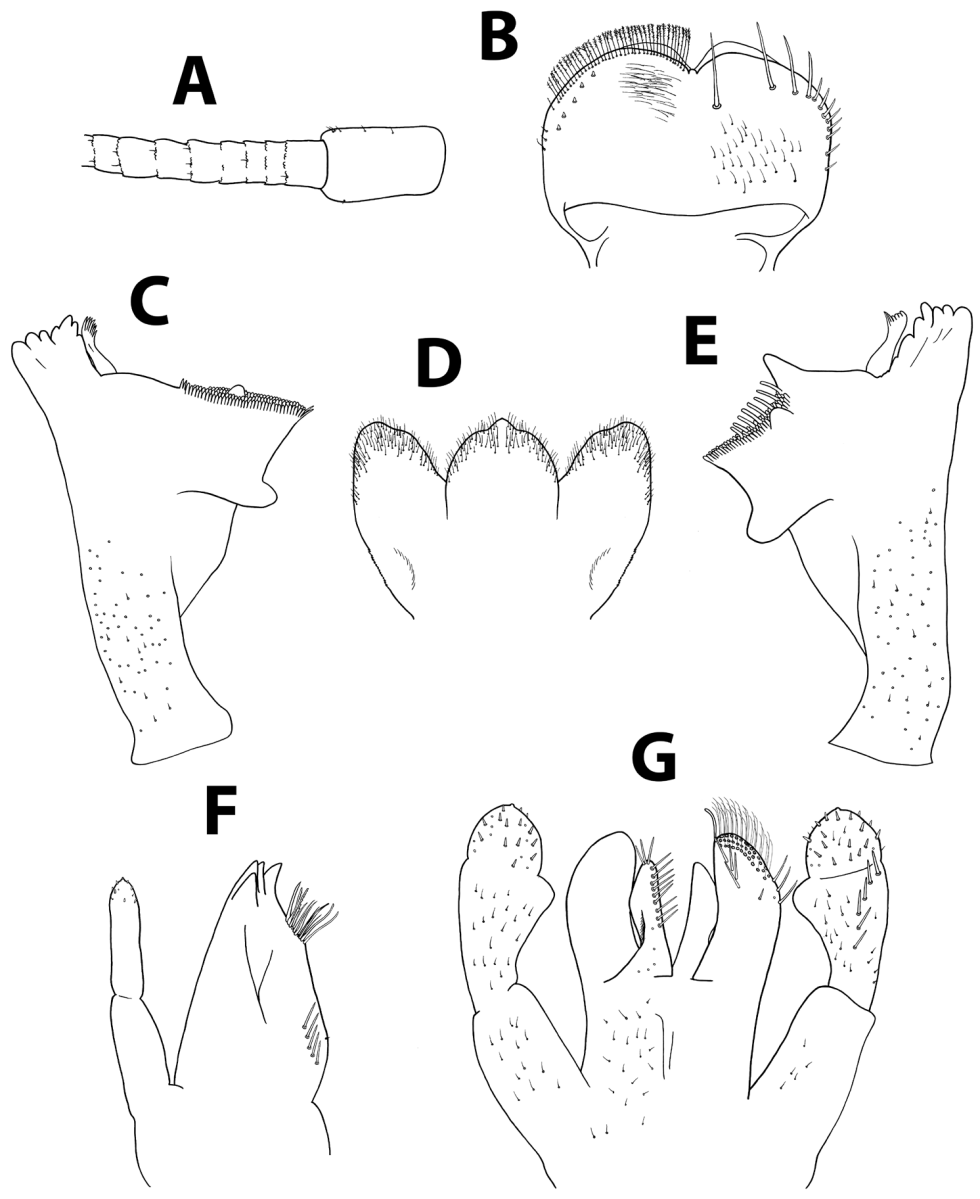
*Baetisgolanensis* sp. n., nymph. **A** antennal pedicel and first flagellomeres **B** labrum (left, ventral; right, dorsal) **C** right mandible **D** hypopharynx **E** left mandible **F** maxilla **G** labium.

*Head.* No carina between antennae; antennae with few fine setae, pedicel without distal lobe (Figure 7A). Labrum (Figure 7B): dorsal surface with scattered, fine, short setae and seta bases, with one median pair of long setae (sometimes with one additional pair of shorter setae); lateral margin with distal arc of 3–4 long, simple, stout setae and 3–8 lateral setae; ventral surface with five small, stout setae laterodistally; distal margin with row of 30–40 fine, long, feathered setae. Hypopharynx (Figure 7D): lingua tri-lobed; surface of lingua and superlingua densely covered with thin setae; lateral margin of superlingua serrated proximally. Right mandible (Figure 7C) with incisors composed of two sets of four and three denticles, all denticles nearly of same size; prostheca with small pointed denticles; space between prostheca and mola without setae or crenulation, mola apex with tuft of setae; base of mandible with many short, thin setae and seta bases. Left mandible (Figure 7E) with incisors composed of six denticles; prostheca with four broad denticles and comb-shaped structure; space between prostheca and mola without setae or crenulation; base of mola without spines; base of mandible with many setae and seta bases. Maxillae (Figure 7F) with four broad teeth; lacinia with one row of small setae and long, serrated setae; base of lacinia with row of five long, stout setae; one seta perpendicular to lacinia margin; palpus 2-segmented, segments I and II subequal; segment II with few minute, thin setae apically, apical nipple with single scale. Labium (Figure 7G): glossa thin, slightly shorter than paraglossa; glossa inner margin and apex with long, stout setae; paraglossa thick, not curved, with 4–5 setae medio-dorsally; paraglossa with apical stout, blunt seta and distal third with three rows of long, feathered setae; palpus 3-segmented; segment I same length as segments II and III combined; segment II with distolateral protuberance slightly developed and dorsal row of 4–6 long, pointed setae; segment III symmetrical, conical, with distinct nipple apically, covered with thick setae. Mentum and palpi covered with many short fine setae ventrally.

**Figure 8.**
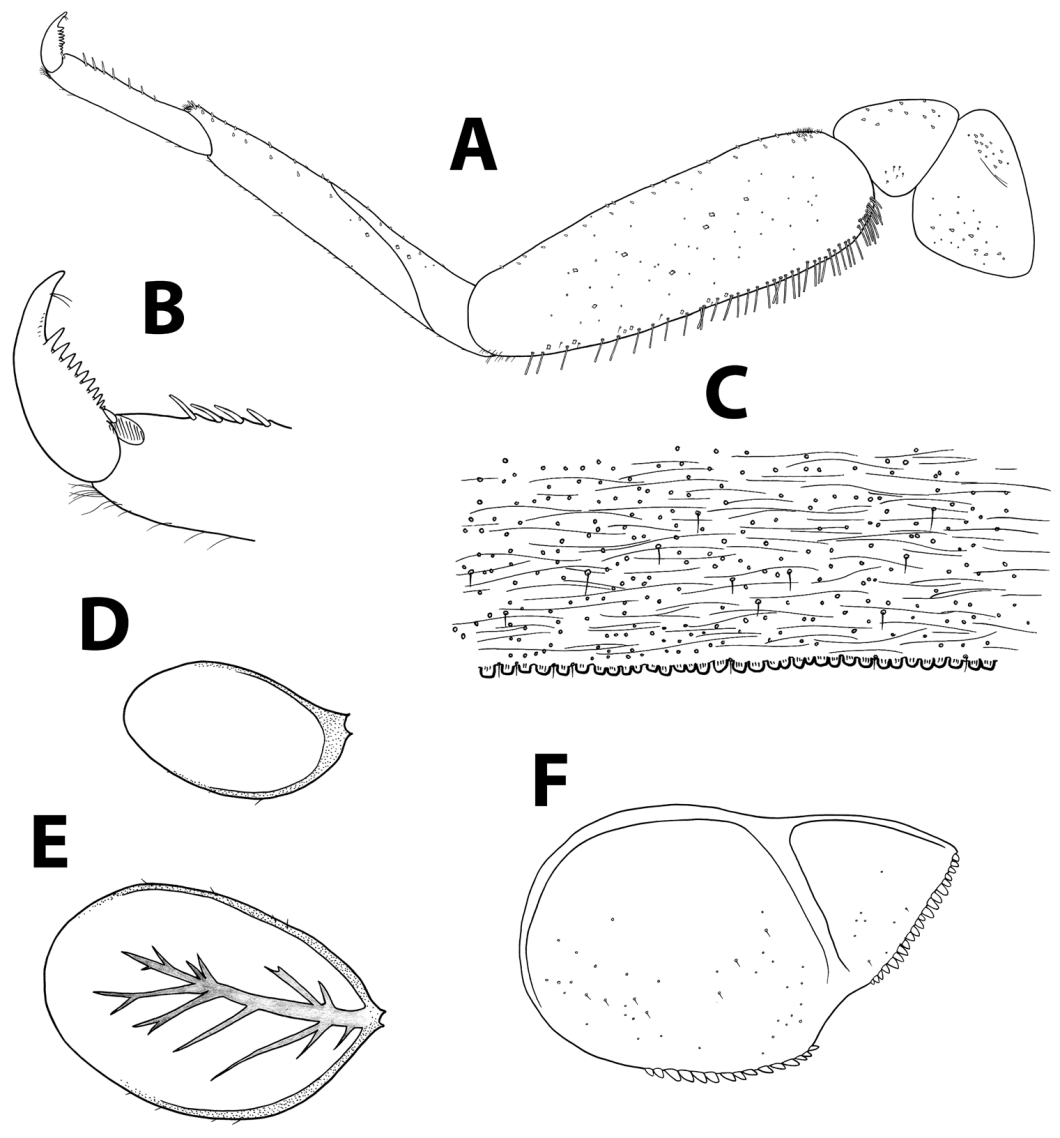
*Baetisgolanensis* sp. n., nymph. **A** foreleg **B** tarsal claw **C** abdominal tergum V, surface and distal margin **D** gill I **E** gill IV **F** paraproct plate.

**Figure 9.**
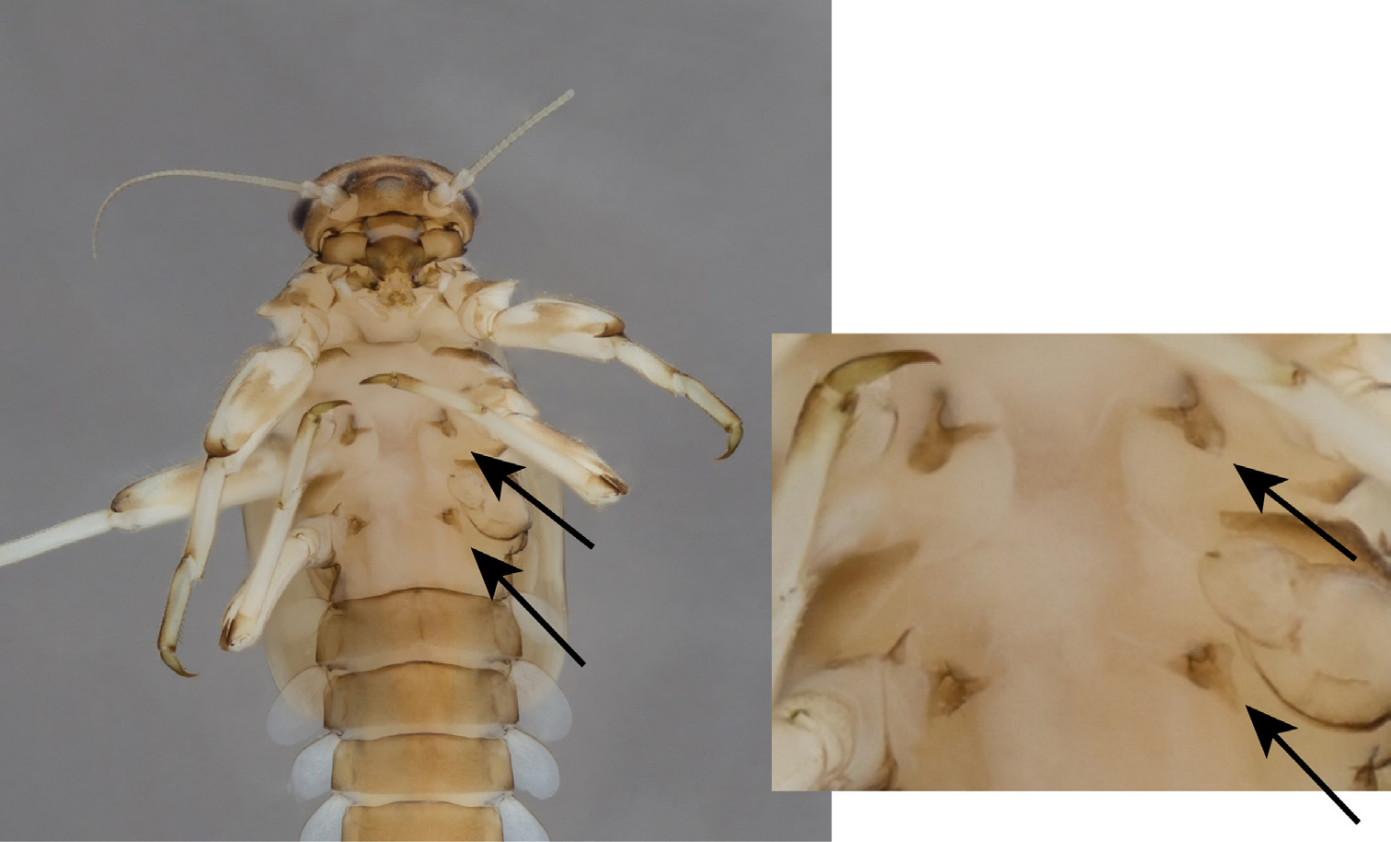
*Baetispacis* sp. n., nymph, ventral, showing protuberances next to coxae (arrows), which are typical to nymphs of *B.pacis* and *B.golanensis*.

*Thorax.* Forelegs (Figure 8A): coxae and trochanters with scattered, minute, stout setae. Femora with dorsal row of 30–40 long, blunt setae, sometimes with few submarginal setae, approximately 1/3 of femur width, with dense group of 5–10 sharper setae proximally; ventral roughly arranged row of 10–20 minute, stout setae; dorsoapical setal patch composed of few thin setae; surface with scattered seta bases and few feathery, brush-like scales, more evident near femur margins. Tibiae with dorsal row of thin, short setae; ventral margin with two rows of sparse minute, stout setae and brush of thin setae apically; tibiopatellar suture present. Tarsi with 5–12 pointed setae ventrally; dorsal margin with row of short, thin setae; one pointed seta, shorter than most ventral setae, at ventral tarsus-claw meeting point. Tarsal claws (Figure 8B) hooked, with row of 8–10 acute teeth and two subapical thin setae (sometimes hardly visible). Mid- and hindlegs similar to forelegs. Round, unsclerotized protuberances, sometimes hardly seen, adjacent to coxa bases (Figure 9).

*Abdomen.* Terga shagreened, with few thin setae and many seta bases, without scales or scale bases; distal margin of all terga with irregular row of blunt, quadrangular spines, with occasional fine seta (Figure 8C), spines absent or rare on terga I and II, more abundant and developed on following terga. Gill I slightly reduced, without visible tracheation (Figure 8D); gills II to VII oval, without marginal spines, main tracheation branched and pigmented (Figure 8E). Paraproct (Figure 8F) surface with few hair-like setae and seta bases; margin with 13–15 short, regular, triangular spines; postero-lateral extension with few seta bases, margin with 18–21 small, triangular spines, sometimes with few submarginal, spatulated scales.

##### Affinities.

The new species clearly belongs to the *Baetislutheri* species group sensu Müller-Liebenau (1969) based on the reduction of the median caudal filament, blunt spines on distal margin of terga, two subapical setae on the claws, and single scale on the apical nipple of the maxillary palp (Bauernfeind and Soldán 2012).

*Baetislutheri* Müller-Liebenau, 1967 appears to be the closest species to *B.golanensis*, based on its morphology, yet differs from it by the incisors of the right mandible, which is variable in size, the arrangement and shape of the setae along the dorsal margin of femur, and the more regular, blunt teeth along the distal margin of the terga (Müller-Liebenau 1967, 1969). In *Baetisvardarensis* Ikonomov, 1962 the ventral protuberances next to the coxae are pointed and more sclerotized than in *B.golanensis* (Müller-Liebenau 1974: fig. 4i; Eiseler 2005: figs 76–77), and *B.vardarensis* has several rows of a smaller number of teeth along the paraproct margin (Ikonomov 1962, Müller-Liebenau 1969). *Baetisestrelensis* Müller-Liebenau, 1974, *B.mirkae* Soldán & Godunko, 2008, *B.nigrescens* Navás, 1932 and *B.pacis* sp. n. can easily be separated from *B.golanensis* by their extremely reduced median caudal filament (Figure 4B vs. Figure 4E). Moreover, *B.mirkae* from Cyprus has many spatulas on body surface, and an additional row of long setae on the proximal part of the dorsal margin of femora, which distinguish it from *B.golanensis*. It also differs from *B.golanensis* and *B.lutheri* by lacking the brush scales on femora (Soldán and Godunko 2008). *Baetismeridionalis* Ikonomov, 1954, which was assigned to the *Baetislutheri* species group by Müller-Liebenau (1969), differs from *B.golanensis* by the lack of scale at the apex of its maxillary palpus, short setae on margin of femora and shape of spines on the distal margin of terga. It is noteworthy that the description of *B.meridionalis* is superficial, it has no type material that could be examined, and its assignment to this species group is uncertain (Soldán and Godunko 2008; Bauernfeind and Soldán 2012).

*Baetislutheri* has a wide distribution in the West Palearctic (Bauernfeind and Soldán 2012) but recent molecular analysis has shown that it may constitute a complex of closely related species (Tenchini et al. 2018). Koch (1988) reported it from Lebanon and Syria, but these reports may refer to *B.golanensis*. *Baetisgolanensis* also corresponds to the undescribed “*Baetis* A12” mentioned by Samocha, also from Israel (1972: 44, pls XVIII–XIX).

##### Etymology.

The species name reflects its presently known distribution range, the Golan Heights.

##### Distribution and ecology.

*Baetisgolanensis* is a rare species in Israel. It was reported by Samocha (as “*Baetis* A12”) from springs and streams in the western Golan and a few localities along the upper Jordan River. This restricted distribution is confirmed in the present study. Two distinct habitats are typical for *B.golanensis* in Israel: streams with large water discharge and rapid currents (upper Jordan River; Figure 2D), and small tributaries with shallow, quiet water running over basaltic pebble substrate (small streams and spring-fed brooks in the Golan Heights; Figure 2E). In all cases, the water is pristine, well oxygenized (>85% dissolved oxygen) and contains aquatic vegetation. In all collecting events of the species, temperatures ranged between 19–25°C and salinity levels between 160–170 ppm. In Israel, mature nymphs were collected mainly in spring and early summer (May–July).

##### Material examined.

**Holotype**. ISRAEL: 1N, Divsha Spring, 33.0901°N, 35.6483°E, ca. 150 m a.s.l., 11.v.2016, Z. Yanai & N. Dorchin, SMNHTAU292000. **Paratypes**. ISRAEL: 14N (3N on slides), Divsha Spring, 10.vi.2014, Z. Yanai; 77N, Divsha Spring, 06.xi.2015, Z. Yanai & S. Cohen; 74N, Divsha Spring, 11.v.2016, Z. Yanai & N. Dorchin; 19N, Divsha Spring, 17.v.2016, Z. Yanai; 3N, Divsha Spring, 02.x.2016, Z. Yanai & N. Truskanov; 16N (1N on slide), Tina Spring, 10.iii.2017, Z. Yanai & J.-L. Gattolliat; 4N, Divsha Spring, 11.iii.2017, Z. Yanai & J.-L. Gattolliat; 44N, Yehudiyya Stream, 11.iii.2017, Z. Yanai & J.-L. Gattolliat. **Other material**. ISRAEL: 1N, Maymon Spring, 22.vi.2014, Z. Yanai; 6N (1N on slide), Gilbon Stream (downstream Gilbon Spring), 15.vii.2014, Z. Yanai; 4N, Divsha Spring, 11.v.2015, Y. Hershkovitz & T. Eshcoly; 1N, Orevim Stream, 11.v.2015, Y. Hershkovitz; 14N, Samakh Stream, 13.v.2015, Y. Hershkovitz & T. Eshcoly; 11N, Jordan River (Ateret Fortress), 29.x.2015, Z. Yanai & Y. Brenner; 7N, Senir Stream (Bet Hillel), 05.xi.2015, Z. Yanai & S. Cohen; 16N, Tina Spring, 06.xi.2015, Z. Yanai & S. Cohen; 2N, Gilbon Stream (upstream Devora Waterfall), 11.v.2016, Z. Yanai & N. Dorchin; 2N, Tina Spring, 16.v.2016, Z. Yanai & A. Charvet; 162N, Jordan River (Ateret Fortress), 16.v.2016, Z. Yanai & A. Charvet; 1N, Senir Stream (Beth Hillel), 17.v.2016, Z. Yanai & A. Charvet; 3N, Jordan River (Neot Mordekhay), 01.vi.2016, Y. Hershkovitz; 8N (2N on slides), Jordan River (Ateret Fortress), 16.x.2016, Z. Yanai.

#### Baetis monnerati

Taxon classificationAnimaliaEphemeropteraBaetidae

Gattolliat & Sartori, 2012

**Figure 10.**
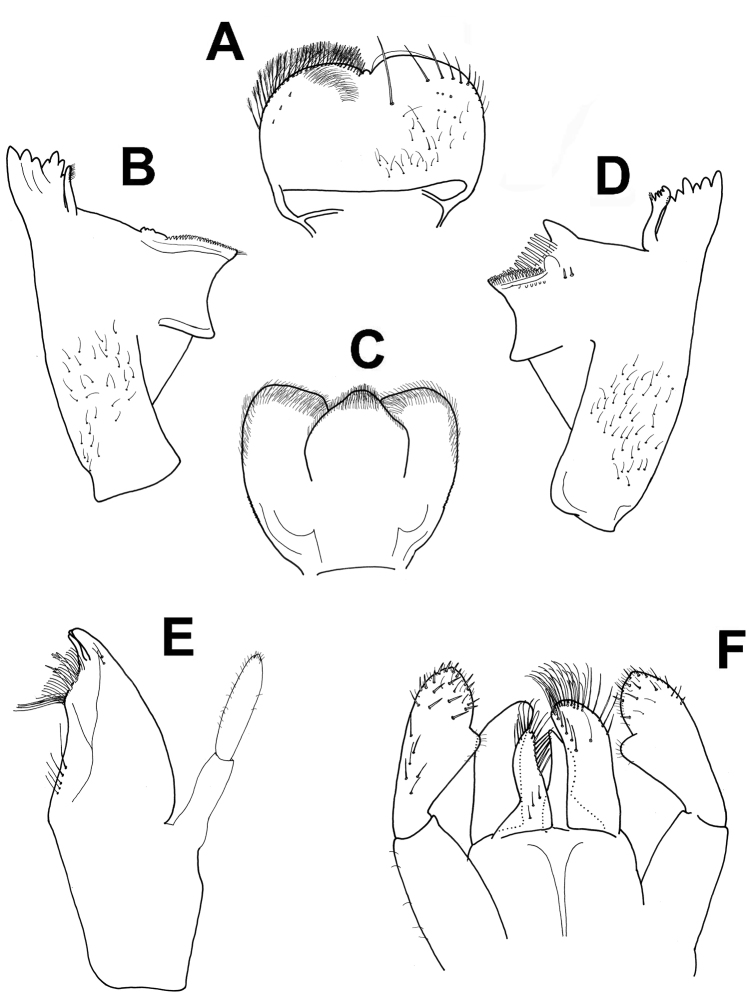
*Baetismonnerati*, nymph. **A** labrum (left, ventral; right, dorsal) **B** right mandible **C** hypopharynx **D** left mandible **E** maxilla **F** labium. Reproduced with permission from Gattolliat et al. (2012).

**Figure 11.**
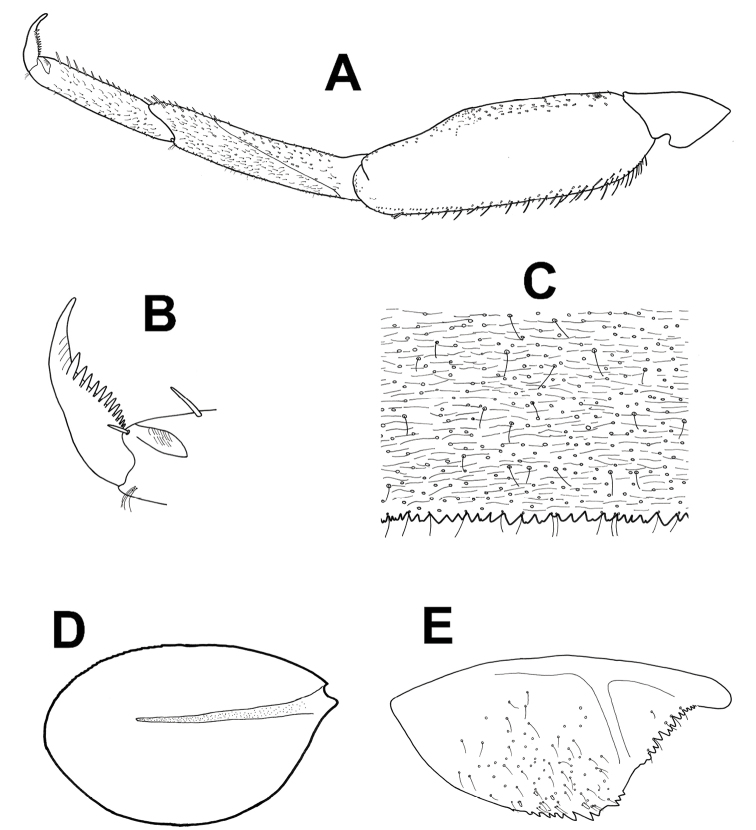
*Baetismonnerati*, nymph. **A** foreleg **B** tarsal claw **C** abdominal tergum V, surface and distal margin **D** gill IV **E** paraproct plate. Reproduced with permission from Gattolliat et al. (2012).

Figures 4C , 10 , 11

Baetis monnerati Gattolliat & Sartori in Gattolliat et al. 2012: 99–104, figs 25–47; Al Hejoj et al. 2014: 366–367, fig 2A; Bojková et al. 2018: 95; Ramadan and Katbeh-Bader 2018: 120, pl 1.

##### Differential diagnosis.

This species (Figures 10, 11) was described in detail by Gattolliat et al. (2012). This is the only Israeli species that exhibits a combination of developed, blunt dorsal setae on the femora, a regular row of short spines on the distal margin of the abdominal terga, and body and gills that are not elongate. The colour pattern on the abdomen is usually clearly visible (Figure 4C), but a similar pattern may occasionally be seen in other species. *Baetismonnerati* populations in various parts of Israel, Jordan and the Palestinian Authority exhibit a wide morphological diversity compared that in congeners, e.g. in size, colour pattern and size of spines on tergite distal margins.

##### Affinities.

*Baetismonnerati* belongs to the *B.buceratus* species group sensu Müller-Liebenau (1969) and exhibits 21 of the 22 characters of the group as defined by Soldán and Godunko (2009). It was discussed in detail and compared to related species by Gattolliat et al. (2012).

##### Distribution and ecology.

*Baetismonnerati* is the most abundant and widespread *Baetis* species in the Israeli fauna. It is common in the Golan Heights, Hula Valley, Upper and Lower Galilee, Jordan Valley, eastern Judean Mountains, and the Dead Sea area. Outside of Israel it is found in Jordan (Gattolliat et al. 2012; Alhejoj et al. 2014; Ramadan and Katbeh-Bader 2018) and Iran (Bojková et al. 2018). Suitable habitats for *B.monnerati* in Israel vary greatly and include brooks and streams of various sizes in temperate and semi-arid regions (Figure 2A–H), usually with pristine water. It is rarely found in slightly disturbed habitats. It is usually found in large numbers and constitutes a major component of the macroinvertebrate community. Dense populations occur in habitats with pebbles and stones together with algae and submerged vegetation. In Israel, adults and mature nymphs were collected in winter and spring (January–May), and were less abundant in fall (October–November).

##### Material examined.

**Holotype.** JORDAN: 1N, Wadi Weida’a, 15.xii.2010, C. Monnerat. **Other material**. ISRAEL: 3N, Fit Spring, 31.v.2014, Z. Yanai; 4N, Zippori Stream (Ras Ali), 02.iii.2015, Z. Yanai; 9N, Qazabiyye Springs, 11.v.2015, Y. Hershkovitz & T. Eshcoly; 86N, Meron Stream (Meron Spring), 12.v.2015, Y. Hershkovitz & T. Eshcoly; 125N, Iyyon Stream (Qiryat Shemona), 12.v.2015, Y. Hershkovitz & T. Eshcoly; 70N, Samakh Stream, 13.v.2015, Y. Hershkovitz & T. Eshcoly; 25N, Jordan River (Ateret Fortress), 29.x.2015, Z. Yanai & Y. Brenner; 97N, Senir Stream (Bet Hillel), 05.xi.2015, Z. Yanai & S. Cohen; 20N (2N on slides), Zippori Stream (Zippori Springs), 07.xi.2015, Z. Yanai & S. Cohen; 19N, Keziv Stream (Hardalit Spring), 07.xi.2015, Z. Yanai & S. Cohen; 4N, Yehudiyya Stream, 27.iii.2016, Y. Hershkovitz; 11N, Iyyon Stream (Qiryat Shemona), 05.iv.2016, Z. Yanai; 66N, Iyyon Stream (nature reserve), 05.iv.2016, Z. Yanai; 340N (1N on slide), Arugot Stream, 09.v.2016, Z. Yanai; 31N, Enan Stream, 17.xi.2016, Z. Yanai & L. Goren; 160N (1N on slide), Dawid Stream, 07.iii.2017, Z. Yanai & J.-L. Gattolliat; 210N, Arugot Stream, 07.iii.2017, Z. Yanai & J.-L. Gattolliat; 100N, Senir Stream (Bet Hillel), 09.iii.2017, Z. Yanai & J.-L. Gattolliat; 130N, Senir Stream (nature reserve), 09.iii.2017, Z. Yanai & J.-L. Gattolliat; 20N, Iyyon Stream (nature reserve), 05.iv.2016, Z. Yanai & J.-L. Gattolliat; 8N, Tina Spring, 10.iii.2017, Z. Yanai & J.-L. Gattolliat; 23N, Hula (nature reserve), 11.iii.2017, Z. Yanai & J.-L. Gattolliat; 250N, Jordan River (Ateret Fortress), 11.iii.2017, Z. Yanai & J.-L. Gattolliat; 34N, Jordan River (Ariq Bridge), 11.iii.2017, Z. Yanai & J.-L. Gattolliat; 38N, Yehudiyya Stream, 11.iii.2017, Z. Yanai & J.-L. Gattolliat; 80N, Daliyyot Stream, 11.iii.2017, Z. Yanai & J.-L. Gattolliat; 4N, Rekhesh Stream (Rekhesh Spring), 04.v.2017, E. Elron. PALESTINIAN AUTHORITY: 23N (1N on slide), Perat Stream (nature reserve), 14.vi.2014, Z. Yanai; 1N (on slide), Perat Stream (nature reserve), 20.vi.2015, Z. Yanai; 9N, Perat Stream (nature reserve), 24.i.2017, Z. Yanai & K. Tamar; 120N, Perat Stream (nature reserve), 08.iii.2017, Z. Yanai & J.-L. Gattolliat; 8N, Pezael Springs, 08.iii.2017, Z. Yanai & J.-L. Gattolliat.

#### Baetis (Rhodobaetis) noa

Taxon classificationAnimaliaEphemeropteraBaetidae

Yanai & Gattolliat sp. n.

http://zoobank.org/E0F3014E-E433-4696-95E9-6D2A47CC5EA1

**Figure 12.**
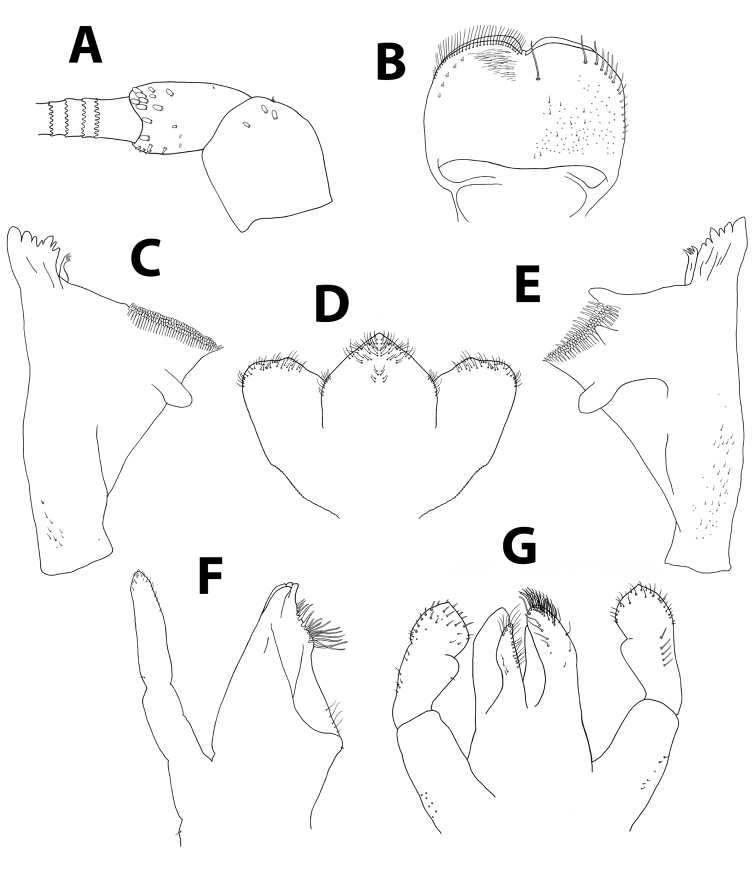
*Baetisnoa* sp. n., nymph. **A** antennal scape, pedicel and first flagellomeres **B** labrum (left, ventral; right, dorsal) **C** right mandible **D** hypopharynx **E** left mandible **F** maxilla **G** labium.

**Figure 13.**
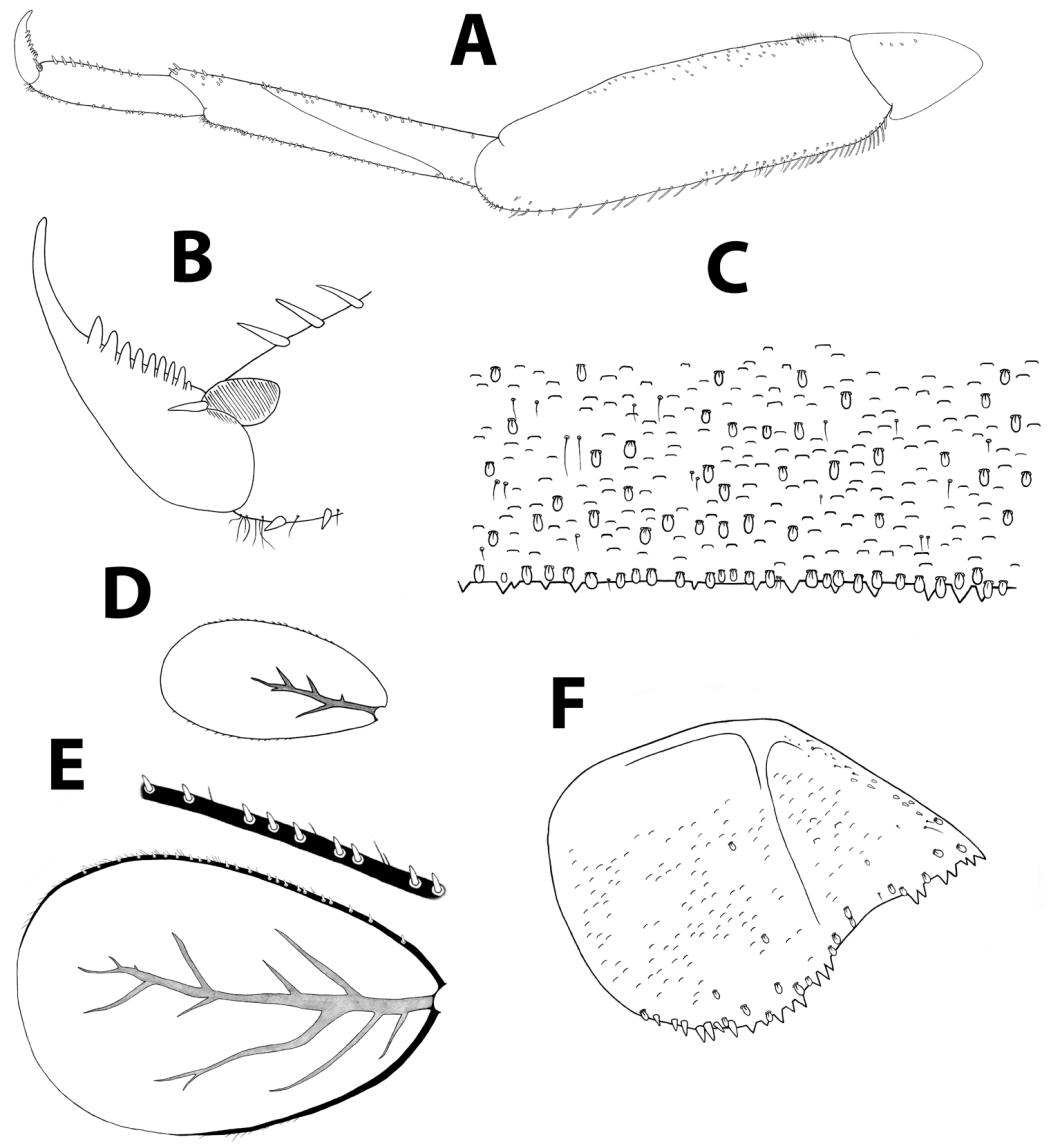
*Baetisnoa* sp. n., nymph. **A** foreleg **B** tarsal claw **C** abdominal tergum V, surface and distal margin **D** gill I **E** gill IV, costal margin, detail **F** paraproct plate.

Figures 4D , 12 , 13

“Baetis L53”: Samocha 1972: 47, pl XX, figs 9–13.

##### Differential diagnosis.

*Baetisnoa* is distinguishable from all known species of *Rhodobaetis* by the following combination of characters: seven setae on labrum, three rows of setae on paraglossae, triangular spines along tergal margins (tergum III and onward), spines on gill margins, ca. 15 triangular teeth along paraproct margin, and 6–8 triangular spines of similar size along cercotractor margin. This is the only representative of *Rhodobaetis* in Israel, and therefore the only one to exhibit the group characteristics, namely body covered by spatulated scales (particularly along the distal margin of the abdominal terga) and stout spines along the gill margins.

##### Description.

*Length* (mature nymphs). Female (n = 24): 4.8–8.1 mm; cerci 1.9–3.0 mm; median caudal filament 1.3–2.3 mm. Male (n = 12): 4.4–6.0 mm; cerci 2.0–2.3 mm; median caudal filament 1.3–1.7 mm.

*Colouration.* General colour brown (Figure 4D). Head brown with ecru spots on frons, around compound eyes and around antennal bases, labrum light brown. Antennae ecru. Turbinate eyes in male nymph amber-brown. Thorax brown with a few pale marks but with no clear pattern. Legs whitish, with light brown spot on dorsal femora, usually with proximal and distal brownish marks on femora and tibiae. Abdominal terga I–IV and VI–VIII brown with fade, pale triangular posteromedial and posterolateral spots. On terga V–IX pale spot larger, covering almost entire posterior half of tergum, with brown margins. Tergum X brown. On all terga a pair of median dark dots occasionally present. Abdominal sterna ecru to light brown. Gills milky, margins dark brown and tracheation violet. Cerci and median caudal filament ecru without bands or pattern.

*Head.* No carina between antennae; pedicel with elongate spatulated scales apically (Figure 12A). Dorsal surface of labrum (Figure 12B) with scattered fine, hair-like setae and seta bases, one median long seta and distolateral arc of setae composed of six long, simple setae; lateral margin with ca. ten small, fine setae; ventral surface with 2–5 small, stout setae laterodistally; distal margin with row of 30–40 fine, long, feathered setae. Hypopharynx (Figure 12D): lingua and superlingua apically covered with minute, stout setae; base of superlingua laterally serrated. Right mandible (Figure 12C) with incisors composed of seven denticles; prostheca with small denticles; space between prostheca and mola without setae or crenulation; mola apex with tuft of setae. Left mandible (Figure 12E) with incisors composed of six denticles; prostheca with four broad denticles and comb-shaped structure; space between prostheca and mola crenulate, without setae; mola apex with tuft of setae. Maxillae (Figure 12F) with four broad teeth; lacinia with row of abundant small setae ending with stouter, longer setae, second row with two serrated, stout dentisetae; base of lacinia with row of 5–6 long, stout setae; one seta perpendicular to lacinia margin; palpus 2-segmented; segment II almost as long as segment I; segment II with sparse thin setae, apical nipple carrying single short, stout scale. Labium (Figure 12G): glossa shorter than paraglossa; glossa inner margin and apex with long, fine setae; paraglossa slightly curved; apex with stout, blunt seta and distal quarter with three rows of long, simple setae; labial palpus 3-segmented; segment I as long as segments II and III combined; segment II with slightly developed distolateral protuberance, and dorsal row of five long, pointed setae; segment III apex with small, thin setae.

*Thorax.* Forelegs (Figure 13A): trochanters almost bare, with few short setae. Femora with dorsal row of ca. 30 mid-sized, apically rounded setae, sparser distally; one submarginal row of fewer, much shorter setae; dorsoapical setal patch composed of ca. seven stout setae and no thin setae; ventral margin with pointed, stout, short setae, distributed mostly on proximal half; surface almost bare. Tibiae with dorsal row of stout setae and few apical thin setae; ventral margin with evenly distributed row of small, pointed setae; tibiopatellar suture present. Tarsi with ca. ten pointed setae ventrally; dorsal row of ca. 20 minute setae; one pointed seta at ventral tarsus-claw meeting point. Tarsal claws (Figure 13B) hooked with one row of 9–13 acute teeth. Mid- and hindlegs similar to forelegs.

*Abdomen.* Terga densely covered with numerous spatulated scales, scale bases and few thin hair-like setae; distal margin of all terga with numerous spatulas, terga I to IV without spines, terga V and VI with few broad, blunt triangular spines (Figure 13C), abundant on terga VII to IX. Gill I narrow and reduced, both margins serrated (Figure 13D); gills II to VII elliptic, costal margin with robust spines, tracheation branched and clearly visible (Figure 13E). Paraproct (Figure 13F) with abundant scale bases and almost no setae, margin with 13–16 broad, triangular spines and submarginal row of spatulas; postero-lateral extension with numerous scale bases, margin with 6–14 triangular spines of similar size, submarginal row of spatulas and lateral row of small setae.

##### Affinities.

*Baetisnoa* belongs to the subgenus Rhodobaetis Jacob, 2003 (*Baetisrhodani* species group sensu Müller-Liebenau 1969) and exhibits the group characteristics, such as the presence of spatulas on the surface of terga, pedicel, paraproct and femora, and gills which generally bear spines along margin (Jacob 2003, Godunko et al. 2004). The morphological attributes of the 22 Palaearctic species in the group have been reviewed by Godunko et al. (2004, 2015), Soldán et al. (2005) and Gattolliat and Sartori (2008).

The Central European species *B.rhodanirhodani* (Pictet, 1843) (*B.rhodanis.s.*) differs from *B.noa* by having long setae on the dorsal margin of femora and not many tergal scales (Gattolliat and Sartori 2008). *Baetisnoa* also differs in the shape of the labial palpus, number of teeth on the claws and spatula density on most of its body surface. The closest relative to *B.noa* geographically is *B.bisri* Thomas & Dia, 1983, which was originally described from Lebanon. Despite the geographical proximity, some important characters distinguish the two species: *B.bisri* has no spines on the gill margins, 5–6 rows of setae on the paraglossae, a different tergal pattern, and a paraproct with less marginal triangular spines and scales of a different shape (Thomas and Dia 1983). *Baetisnoa* also has a wider labrum compared to these species. Other than *B.bisri* and *B.rhodani*, two more *Rhodobaetis* species were reported from Turkey: *B.braaschi* Zimmermann, 1980, and *B.vadimi* Godunko, Palatov & Martynov, 2015 (Kazancı and Türkmen 2016; Salur et al. 2016). *Baetisgemellus* Eaton, 1885 and *B.pseudogemellus* Soldán, 1977 are excluded, as explained by Godunko et al. (2015). Unlike *B.noa*, no spines are present on the gill margins of *B.braaschi* (Bauernfeind and Soldán 2012) and *B.vadimi* (Godunko et al. 2015). *Baetismilani* Godunko, Prokopov & Soldán, 2004 from the Crimean Peninsula has no triangular spines on the posterior margin of terga and very abundant minute denticles on its cercotractor (Godunko et al. 2004). The North African *B.sinespinosus* differs from *B.noa* by having more submarginal setae on the labrum, four setal rows on the paraglossa, fewer tergal scales, and considerably less marginal teeth on the paraproct (Soldán et al. 2005). *Rhodobaetis* was also reported from Cyprus by Soldán and Godunko (2008), who described a new species, *B.irenkae* Soldán & Godunko, 2008, presumably endemic to the island. *Baetisirenkae* differs from *B.noa* by the shape of its labial palpus, shape and abundance of spatulas on the distal margin of terga, and number and shape of spines on the margin of cercotractor. The most similar species to *B.noa* morphologically is *B.tauricus* Godunko & Prokopov, 2003 which is found exclusively in the Crimean Peninsula. However, the two species differ by the number and size of the spines along the margin of the cercotractor.

*Baetisnoa* matches the characters of the undescribed “*Baetis* L53” as outlined in Samocha (1972; p. 47 and plate XX therein). In his review of the Israeli mayfly fauna, this was the only species that had spines on its gill margins and a nipple on the apex of its maxillary palpus. It also matches in colour and distribution.

##### Etymology.

The name is a noun in apposition. The species is dedicated to Noa Truskanov, the partner, source of inspiration, and closest friend of the senior author.

##### Distribution and ecology.

This species is known from the tributaries of the Jordan River and from brooks and springs in the Golan Heights, where it inhabits streams of diverse sizes and current velocities (Figs 2A, E). Typical substrate in its habitats is composed mainly of pebbles and rocks of various sizes, as well as rich epilithic and submerged vegetation. Mature nymphs were collected mostly in spring (March–May), but also in fall (October–November).

##### Material examined.

**Holotype**. ISRAEL: 1N, Senir Stream (nature reserve), 33.2331°N, 35.6223°E, ca 130 m a.s.l., 09.iii.2017, Z. Yanai & J.-L. Gattolliat, SMNHTAU292002. **Paratypes**. ISRAEL: 2N, Senir Stream (Bet Hillel), 09.vi.2014, Z. Yanai; 3N (1N on slide), Senir Stream (nature reserve), 15.vii.2014, Z. Yanai; 3N, Dan Stream (Dafna), 29.vii.2015, Z. Yanai; 7N, Dan Stream (Tel Dan), 05.xi.2015, Z. Yanai & S. Cohen; 2N (on slides), Senir Stream (nature reserve), 05.xi.2015, Z. Yanai & S. Cohen; 6N, Dan Stream (Tel Dan), 31.v.2016, Y. Hershkovitz; 68N, Dan Stream (Dan), 31.v.2016, Y. Hershkovitz; 24N (1N on slide), Senir Stream (nature reserve), 09.iii.2017, Z. Yanai & J.-L. Gattolliat; 80N, Dan Stream (Tel Dan), 10.iii.2017, Z. Yanai & J.-L. Gattolliat; 2N, Dan Stream (Dan), 07.v.2017, Z. Yanai; 13N, Yehudiyya Stream, 12.iv.2018, Z. Yanai; 36N, Dan Stream (Dafna), 13.iv.2018, Z. Yanai. **Other material**. ISRAEL: 8N (1N on slide), Hermon Stream (Panyas Springs), 19.ii.2014, Z. Yanai; 16N (1N on slide), Maymon Spring, 22.vi.2014, Z. Yanai; 18N, Senir Stream (nature reserve), 15.vii.2014, Z. Yanai; 3N, Senir Stream (Bet Hillel), 05.xi.2015, Z. Yanai & S. Cohen; 54N, Maymon Spring, 04.iv.2016, Z. Yanai; 10N, Dan Stream (Dafna), 31.v.2016, Y. Hershkovitz.

#### Baetis pacis

Taxon classificationAnimaliaEphemeropteraBaetidae

Yanai & Gattolliat sp. n.

http://zoobank.org/BE255364-B1D4-4A80-8407-CC1A8CA8591D

**Figure 14.**
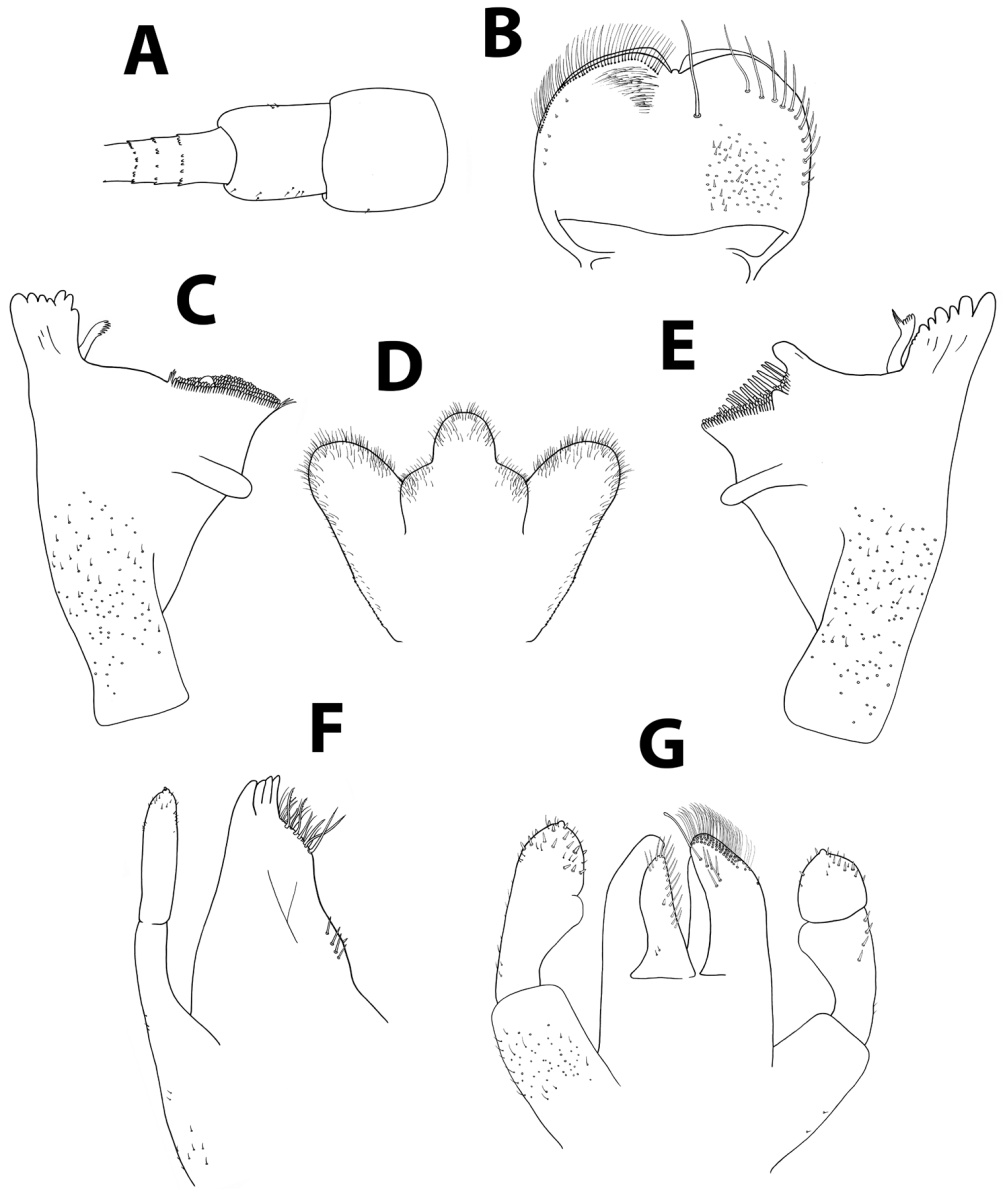
*Baetispacis* sp. n., nymph. **A** antennal scape, pedicel and first flagellomeres **B** labrum (left, ventral; right, dorsal) **C** right mandible **D** hypopharynx **E** left mandible **F** maxilla **G** labium.

**Figure 15.**
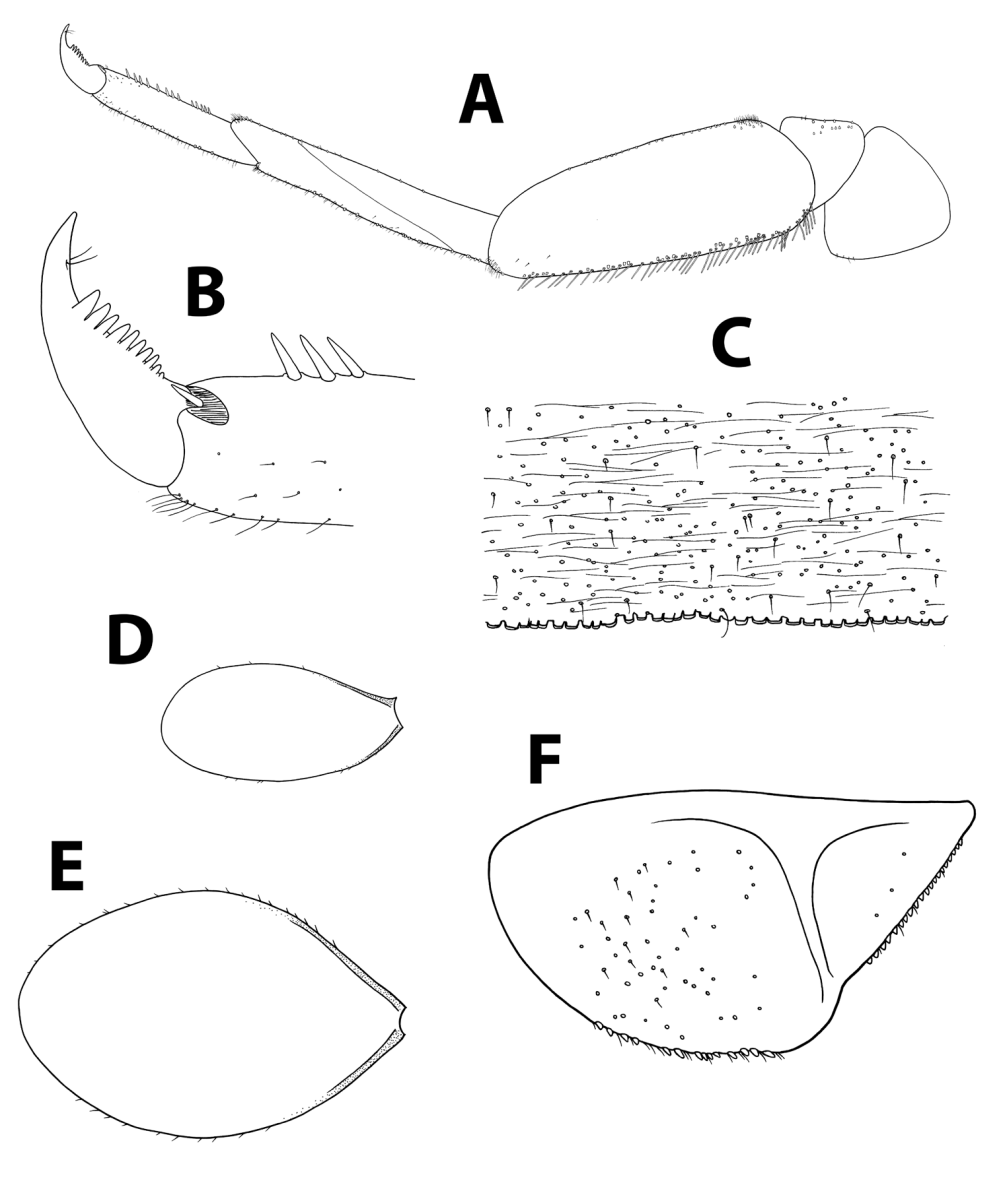
*Baetispacis* sp. n., nymph. **A** foreleg **B** tarsal claw **C** abdominal tergum V, surface and distal margin **D** gill I **E** gill IV **F** paraproct plate.

Figures 4E , 14 , 15

“Baetis L12”: Samocha 1972: 45, pl XIX, fig. 10.
“Baetis sp.”: Alhejoj et al. 2014: 352, fig. 2B.

##### Differential diagnosis.

Within the *Baetislutheri* species group, *B.pacis* is distinguishable by the following combination of characters: greatly reduced median caudal filament, non-sclerotized protuberances next to coxae, and body not covered with spatulae. In Israel it is easily recognized by its very short median caudal filament.

##### Description.

*Length* (mature nymphs). Female (n = 13): 5.0–7.6 mm; cerci 2.7–4.5 mm; median caudal filament minute (< 0.2 mm). Male (n = 10): 4.0–6.0 mm; cerci 2.2–4.1 mm; median caudal filament minute (< 0.2 mm). Usually, specimens from the Perat Stream population are smaller than those in northern Israel.

*Colouration.* General colour brown (Figure 4E). Head brown with light vermiform mark, antennae ecru. Turbinate eyes in male nymph amber. Thorax brown. Legs ivory to whitish, claws, apex of femora and tibiae dark brown, dorsal face of femora with medium brown central mark. Abdominal terga light brown with two central dark brown spots more or less expanded, generally also darker laterally and distally, terga V, VI, IX and X brighter. Abdominal sterna ecru to pale brown. Gills milky, semi-transparent, almost no visible tracheation. Cerci ecru to light brown, without bands or pattern.

*Head.* No carina between antennae; pedicel almost bare, without distal lobe (Figure 14A). Labrum (Figure 14B): dorsal surface with scattered fine, short setae and seta bases, with one median pair of long setae; lateral margin with distal arc of five long, simple, stout setae and 6–8 lateral setae; ventral surface with 5–6 small, stout setae laterodistally; distal margin with a row of 30–50 fine, long, feathered setae. Hypopharynx (Figure 14D): lingua tri-lobed; surface of lingua and superlingua densely covered with medium thin setae; lateral margin of superlingua serrated proximally. Right mandible (Figure 14C) with incisors composed of two sets of four and three denticles, outer denticles of each set more prominent; prostheca with small, pointed denticles; space between prostheca and mola without setae or crenulation; mola apex with tuft of setae; base of mandible with many short, thin setae and seta bases. Left mandible (Figure 14E) with incisors composed of 6 denticles, inner denticle with additional minute teeth; prostheca with 4–5 broad denticles and comb-shaped structure; space between prostheca and mola without setae or crenulation; base of mola with no spines; base of mandible with many setae and seta bases. Maxillae (Figure 14F) with four broad teeth; lacinia with one row of small setae and long, serrated setae; base of lacinia with row of four long, stout setae; one seta perpendicular to lacinia margin; palpus 2-segmented, segments I and II subequal; segment II with few minute, thin setae apically, and apical nipple with single scale. Labium (Figure 14G): glossa shorter than paraglossa; inner margin and apex of glossa with medium, stout setae; paraglossa not curved, dorsal side with inner 4–6 setae; apical stout, blunt seta; paraglossa apex and distal third with four rows of long, simple setae; labial palpus 3-segmented; segment I similar in length to segments II and III combined; segment II with distolateral protuberance slightly developed, and dorsal row of 4–5 long pointed setae; conic segment III symmetrical, with apical distinct nipple, covered with needle-shaped blunt setae.

*Thorax.* Forelegs (Figure 15A): coxae almost bare. Trochanters with few ventral minute, stout setae. Femora with one or two dorsal rows with total of ca. 40 stout setae, no longer than 1/4 of femur width, denser proximally; ventral row of ca. 20 minute, stout setae; dorsoapical setal patch made of maximum five minute setae and few thin setae; surface almost bare. Tibiae with dorsal row of thin, short setae and few scales; ventral margin with minute, stout setae and apical brush of thin setae; tibiopatellar suture present. Tarsi with 6–13 ventral pointed setae; dorsal margin with row of short, thin setae; one pointed seta, shorter than most ventral setae, at ventral tarsus-claw meeting point. Tarsal claws (Figure 15B) hooked, with one row of 7–11 acute teeth and two subapical thin setae. Dorsal margin of femora, tibiae and tarsi with scattered feathery scales. Mid- and hindlegs similar to forelegs. Round, unsclerotized protuberances, sometimes hardly seen, adjacent to coxa bases (Figure 9).

*Abdomen.* Terga shagreened with few thin setae and many seta bases, without scales; distal margin with row of blunt quadrangular spines (Figure 15C). Gills I to VII oval, without marginal spines, main tracheation not pigmented and poorly branched (on gills II to VI main trachea sometimes slightly visible; Figures 15D, E); gills I and VII slightly reduced. Paraproct (Figure 15F) surface with few hair-like setae and seta bases; margin with 10–15 short triangular spines and short thin setae; postero-lateral extension with seta bases, margin with 15–20 small triangular spines. Median caudal filament extremely short, approximately ten segments long.

##### Affinities.

The species belongs to the *Baetislutheri* species group sensu Müller-Liebenau (1969) based on its morphological characteristics (see details under *B.golanensis* above).

*Baetislutheri* differs from *B.pacis* by having longer setae on the dorsal margin of femora, densely shagreened terga surface and less reduced median caudal filament (Müller-Liebenau 1969). *Baetismirkae* from Cyprus differs from *B.pacis* by having wider labrum with fewer dorsal long setae, only three rows of setae on the paraglossae, broader labial palpus, more setae on the femora and tibiae, terga not shagreened, rounded teeth on distal margin of terga, and generally more spatulas and setae on the body surface than in *B.pacis* (Soldán and Godunko 2008). The European species *B.vardarensis* is characterized by having unique sclerotized, pointed protuberances next to the coxae (Müller-Liebenau 1974: fig. 4i; Eiseler 2005: figs 76–77), which, in the case of *B.pacis*, are rounded and not sclerotized, exhibiting the more common situation in the group. Moreover, *B.vardarensis* has a hypopharynx with a uni-lobed lingua, and dorsal line of long setae on the femur margin (Ikonomov 1962). Within the *lutheri* species group, *B.pacis*, *B.estrelensis*, *B.mirkae* and *B.nigrescens* can be distinguished by their greatly reduced median caudal filament, only a few segments long, while other species have a medial caudal filament which is at least as long as 1/4 (*B.vardarensis*) or 1/2 (*B.golanensis*, *B.lutheri*, *B.meridionalis*) of the cerci. The number of rows of long setae on the proximal part of the dorsal margin of femora varies among species: one row in *B.lutheri* and *B.golanensis*, 2–3 rows in *B.mirkae*, and 4–5 rows in *B.vardarensis* (Müller-Liebenau 1974: fig. 5a, b; Soldán and Godunko 2008). *Baetispacis* generally has one or two rows of setae, with occasional additional setae that may appear as a third row. Variations can be observed between specimens and even between legs of the same individual. *Baetispacis* clearly differs from *B.estrelensis* and *B.nigrescens* by the shape of the labial palp (much slenderer in *B.estrelensis and B.nigrescens*), and the much less shagreened surface of the abdominal terga (Müller-Liebenau 1971, 1974).

This species corresponds to the undescribed “*Baetis* L12” of Samocha (1972: 45 and pl XIX) and probably also to “*Baetis* sp.” mentioned by Alhejoj et al. (2014) from Jordan.

##### Etymology.

This species occurs in Israel, the Palestinian Authority, and apparently also in Jordan. Its name reflects our yearning for regional peace.

##### Distribution and ecology.

*Baetispacis* is distributed mainly in the tributaries of the Jordan River (i.e., Senir, Dan and Iyyon; Figure 2A) but is occasionally encountered in small numbers in smaller Golan streams (Figure 2E) and in the vicinity of the Sea of Galilee (e.g., Arbel Stream). Based on details in Samocha (1972), it may also occur in the Hermon (Panyas) and Ammud streams. An isolated population is found in Perat Stream (Wadi Qelt; Figure 2G). Alhejoj et al. (2014) referred to “*Baetis* sp.” from Wadi Hisban in Jordan, about 50km east of Perat Stream. Additional study may confirm that this population indeed belongs to *B.pacis*. The Israeli populations probably represent the southeastern limit of distribution for the *B.lutheri* species group. The distribution of *B.pacis* is limited to small- and medium-sized streams with moderate water flow and stony substrate. While water temperature in its habitats may vary, low salinity (< 300 ppm) and high oxygen concentration (>85%) are more important for the species. Mature nymphs were found mostly in spring (April–May) and fall (October–November).

##### Material examined.

**Holotype**: ISRAEL: 1N, Senir Stream (nature reserve), 33.2331°N, 35.6223°E, ca 130 m a.s.l., 09.iii.2017, Z. Yanai & J.-L. Gattolliat. SMNHTAU292001.–**Paratypes**. ISRAEL: 80N (1N on slide), Senir Stream (nature reserve), 09.iii.2017, Z. Yanai & J.-L. Gattolliat; 6N, Iyyon Stream (nature reserve), 05.iv.2016, Z. Yanai; 30N (1N on slide), Senir Stream (nature reserve), 17.v.2016, Z. Yanai & A. Charvet; 13N (1N on slide), Dan Stream (Dan), 31.v.2016, Y. Hershkovitz; 2N, Dan Stream (Dafna), 31.v.2016, Y. Hershkovitz; 16N, Senir Stream (Bet Hillel), 09.iii.2017, Z. Yanai & J.-L. Gattolliat; 1N, Dan Stream (Tel Dan), 10.iii.2017, Z. Yanai & J.-L. Gattolliat. **Other material**. ISRAEL: 1N, Divsha Spring, 06.xi.2015, Z. Yanai & S. Cohen; 1N, Divsha Spring, 11.v.2016, Z. Yanai & N. Dorchin; 1N, Iyyon Stream (nature reserve), 01.vi.2016, Y. Hershkovitz; 1N, Divsha Spring, 02.x.2016, Z. Yanai & J.-L. Gattolliat. –PALESTINIAN AUTHORITY: 1N, Perat Stream (nature reserve), 20.vi.2015, Z. Yanai; 37N, Perat Stream (nature reserve), 11.xi.2015, Z. Yanai & S. Cohen; 22N, Perat Stream (nature reserve), 23.v.2016, Z. Yanai & L. Friedman; 6N (2N on slides), Perat Stream (nature reserve), 24.i.2017, Z. Yanai & K. Tamar; 2N, Perat Stream (nature reserve), 30.i.2017, Z. Yanai & N. Truskanov; 12N, Perat Stream (nature reserve), 08.iii.2017, Z. Yanai & J.-L. Gattolliat.

#### Baetis samochai

Taxon classificationAnimaliaEphemeropteraBaetidae

Koch, 1981

**Figure 16.**
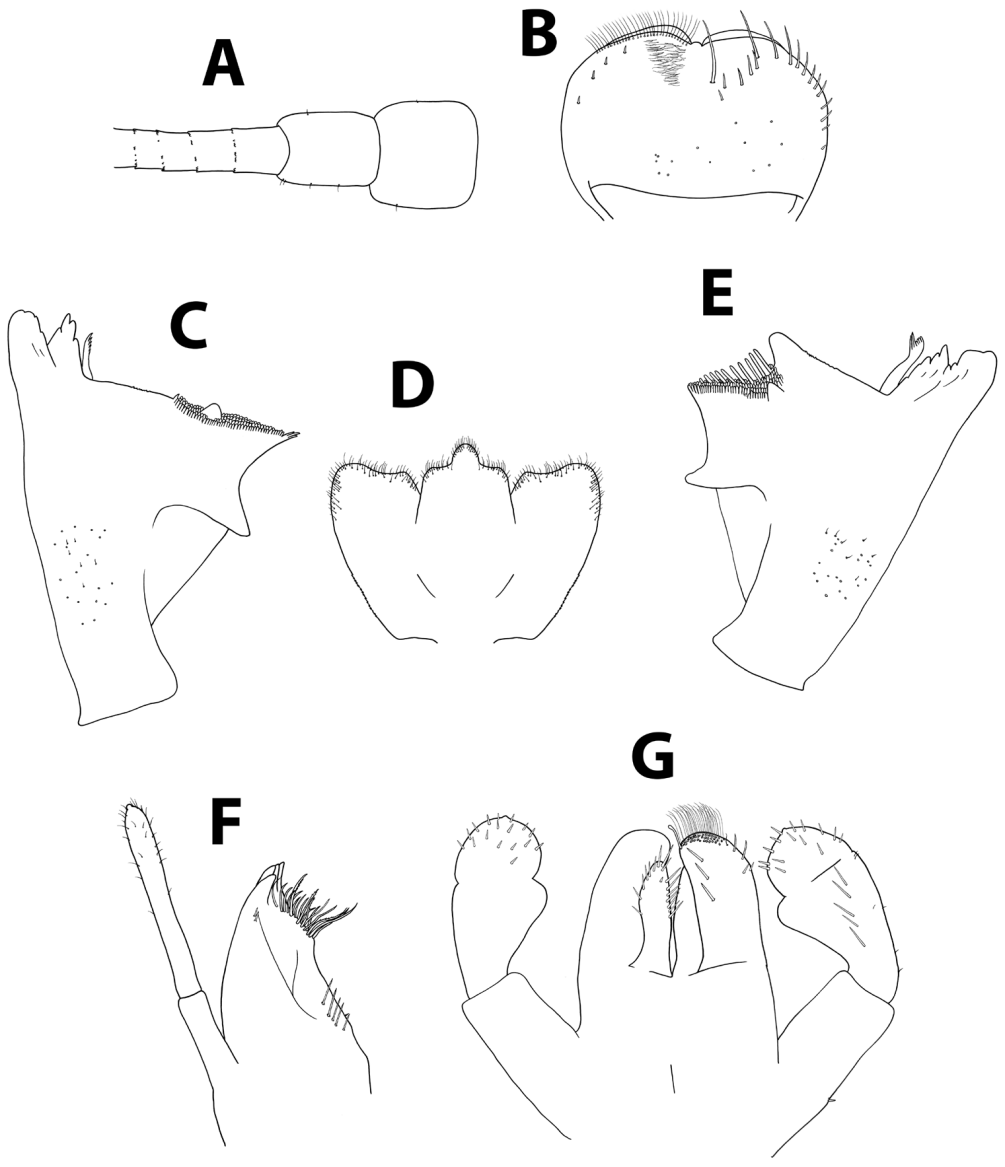
*Baetissamochai*, nymph. **A** antennal scape, pedicel and first flagellomeres **B** labrum (left, ventral; right, dorsal) **C** right mandible **D** hypopharynx **E** left mandible **F** maxilla **G** labium.

**Figure 17.**
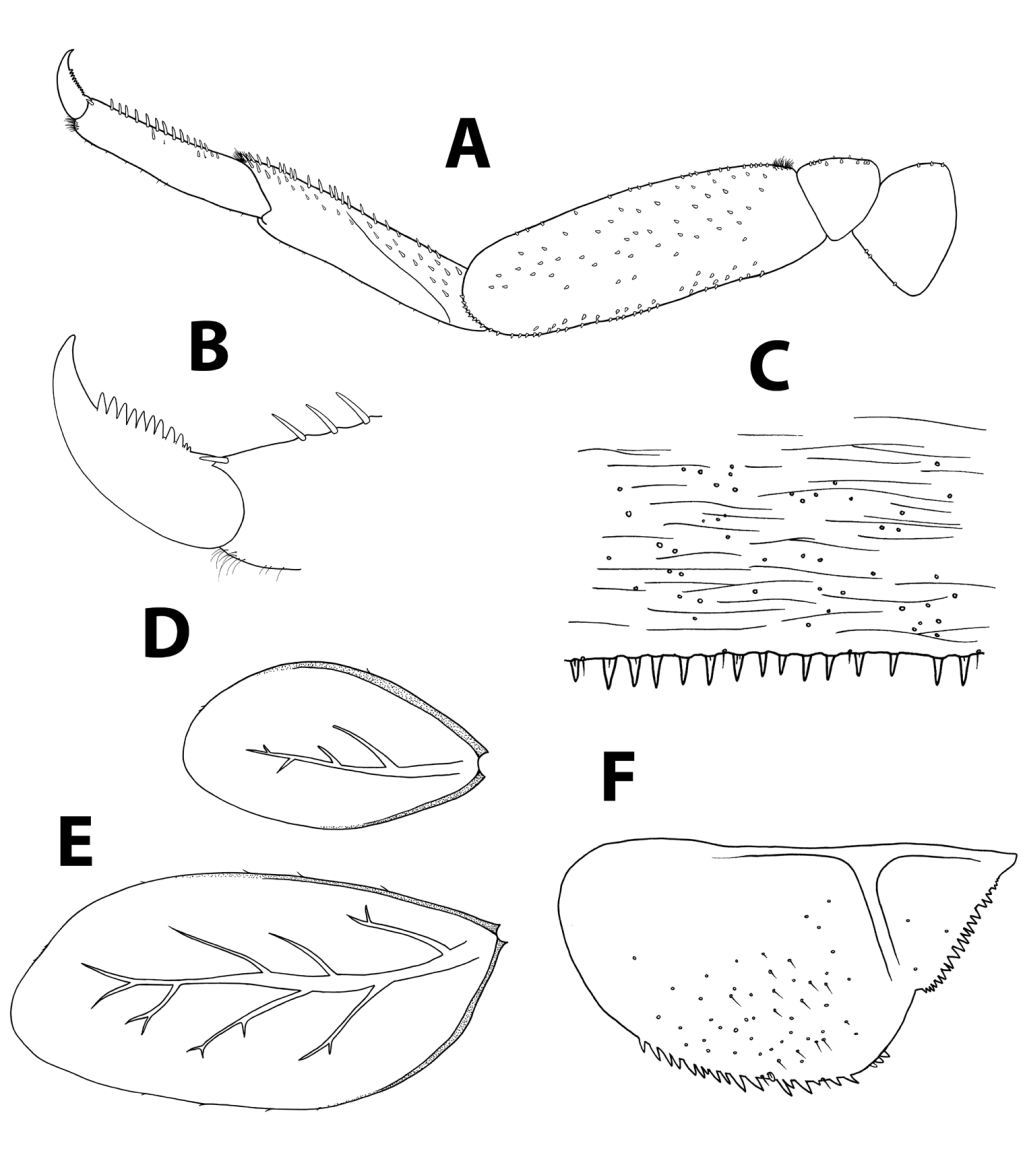
*Baetissamochai*, nymph. **A** foreleg **B** tarsal claw **C** abdominal tergum V, surface and distal margin **D** gill I **E** gill IV **F** paraproct plate.

Figures 4F , 16 , 17

“Baetis L34”: Samocha 1972: 6, pl XX, figs 1–8.
Baetis samochai : Koch 1981: 121–128, figs 1–14; Koch 1988: 94; Tanatmış 1999; Kazancı 2001; Salur et al. 2016: 74; Bojková et al. 2018: 95.

##### Notes.

As the original description (Koch 1981) was superficial, lacked important diagnostic characters, and some of the drawings were of poor quality, we provide a more detailed description below.

##### Differential diagnosis.

*Baetissamochai* is well distinguishable by the following combination of characters: canines of both mandibles with a very broad outer tooth; maxillary palp longer than galea-lacinia; dorsal margin of femora with abundant small, strong setae; elongate body; long gills (about twice the length of following abdominal segment); distal margin of terga with needle-like spines; abdominal colouration yellowish brown with whitish, central longitudinal stripe.

##### Description.

*Length* (of full-grown specimens). Female (n = 22): 6.0–8.2 mm; cerci 4.0–6.5 mm; median caudal filament 2.6–3.7 mm. Male (n = 14): 5.6–7.5 mm; cerci 3.1–6.1 mm; median caudal filament 2.2–3.2 mm.

*Colouration.* General colour yellowish brown with longitudinal stripe on entire body (Figure 4F; Koch 1981: fig. 12).

*Head.* Dorsal surface of labrum (Figure 16B; fig. 5 in Koch 1981) with scattered fine setae and seta bases, one median pair of long setae and distolateral arc of setae composed of 7–8 long, simple, stout setae; lateral margin with 4–6 small, fine setae; ventral surface with 4–5 small, stout setae laterodistally; distal margin with row of ca. 30 fine, long, feathered setae. Right and left mandibles with two sets of denticles relatively deeply cleft (Figure 16C, E; Koch 1981: figs 6, 7), outer denticle very broad, constituting most of outer set. Maxilla (Figure 16F; Koch 1981: fig. 10) with four broad teeth; lacinia with two rows of setae, one row with abundant small setae ending with stouter and longer setae, second row with two serrated, stout dentisetae; base of lacinia with row of five stout setae; one seta perpendicular to lacinia margin; palpus 2-segmented, segment II longer than segment I, exceeding galea-lacinia in length; segment II with thin setae and apical nipple, apical scale absent. Labium (Figure 16G): palpus 3-segmented; segment II with slightly developed distolateral protuberance; segment III symmetrical, rounded.

*Thorax.* Legs (Figure 17A; Koch 1981: fig. 11): Dorsal margin of femora with many scattered short, stout setae.

*Abdomen.* Distal margin of terga with slender triangular spines, more than twice longer than broad (Figure 17C; space between spines wider than originally illustrated by Koch 1981: fig. 10). Gills elongate with developed tracheation (Figure 17D, E; Koch 1981: fig. 14); gill IV twice as long as following tergum. Paraproct (Figure 17F) with abundant seta bases, few hair-like setae and rarely pointed submarginal spatulas; margin with 16–19 long, triangular spines; postero-lateral extension with fewer seta bases, margin with 15–20 triangular spines almost as long as those of paraproct.

##### Affinities.

As discussed by Koch (1981), this species belongs to the *Baetisvernus* species group sensu Müller-Liebenau (1969), although the median caudal filament is clearly shorter than the cerci. This species corresponds to the undescribed “*Baetis* L34” sensu Samocha (1972: 46, pl XX).

##### Distribution and ecology.

*Baetissamochai* is known from Israel, Lebanon, Syria (Koch 1981, 1988), Turkey (Tanatmış 1999; Kazancı 2001; Salur et al. 2016), and Iran (Bojková et al. 2018). In Israel it was recorded by Samocha (1972) exclusively from the northern Golan Heights, but we found numerous populations throughout the Golan Heights, Hula Valley, and the Upper Jordan River and its tributaries. This species may have been overlooked by Samocha or has become more abundant since his study. Unlike most *Baetis* species, *B.samochai* was reported from lotic as well as lentic habitats, with abundant aquatic vegetation (Koch 1981). Indeed, we found this species in Israel in diverse ecological niches, including ponds, marshes and streams with moderate or rapid currents (Figure 2A, B, D, E). It is most typically found in calm microhabitats with abundant vegetation along waterbody margins. Mature nymphs were found mostly in spring (March–May), whereas adults were found in winter (November–December), suggesting that the species has at least two generations per year.

##### Material examined.

ISRAEL: 6N, Senir Stream (nature reserve), 11.iii.2015, Z. Yanai; 2N, Daliyyot Stream, 26.iii.2014, Z. Yanai; 1N, Hermon Stream (Panyas Springs), 09.vi.2014, Z. Yanai; 1N, Jordan River (haPeqaq Bridge), 10.vi.2014, Z. Yanai; 6N, Keziv Stream (Hardalit Spring), 17.vi.2014, Z. Yanai; 1N (on slide), Senir Stream (nature reserve), 15.vii.2014, Z. Yanai; 5N, Hula (nature reserve), 01.xii.2014, Z. Yanai; 11N, Jordan River (haHamisha Bridge), 08.xii.2014, Z. Yanai; 3N, Enan Stream, 29.iv.2015, L. Goren; 3N (1N on slide), Hula (nature reserve), 29.iv.2015, L. Goren; 1N, Rezaniyya Winter Pool, 29.iv.2015, L. Goren; 1N, Qazabiyye Springs, 29.iv.2015, L. Goren; 3N, haKefar Spring, 11.viii.2015, E. Elron; 87N (2N on slides), Gamla Stream (Peham Springs), 28.iii.2016, Y. Hershkovitz; 6N (1N on slide), Gamla Stream (Peham Springs), 04.iv.2016, Z. Yanai; 21N (1N on slide), Ayit Stream (Ayit Waterfall), 04.iv.2016, Z. Yanai; 1N, El-Mahfi Winter Pool, 20.iv.2016, L. Goren; 6N, Qazabiyye Springs, 20.iv.2016, L. Goren; 3N, Dan Stream (Dan), 20.iv.2016, L. Goren; 1N, Divsha Spring, 11.v.2016, Z. Yanai & N. Dorchin; 6N (1N on slide), Jordan River (Ateret Fortress), 16.v.2016, Z. Yanai & A. Charvet; 2N, Jordan River (Neot Mordekhay), 01.vi.2016, Y. Hershkovitz; 6N, Hula (nature reserve), 16.xi.2016, Z. Yanai & L. Goren; 14N, Enan Stream, 17.xi.2016, Z. Yanai & L. Goren; 61N, Senir Stream (nature reserve), 09.iii.2017, Z. Yanai & J.-L. Gattolliat; 7N, Jordan River (Ariq Bridge), 11.iii.2017, Z. Yanai & J.-L. Gattolliat; 1N, Yehudiyya Stream, 11.iii.2017, Z. Yanai & J.-L. Gattolliat; 1N, Ayit Stream (Ayit Waterfall), 11.iii.2017, Z. Yanai & J.-L. Gattolliat; 190N, Hula (nature reserve), 11.iii.2017, Z. Yanai & J.-L. Gattolliat; 41N, Hula (nature reserve), 08.v.2017, L. Goren; 2N, Yehudiyya Stream, 12.iv.2018, Z. Yanai; 1N, El-Muayer Winter Pool, 25.iv.2018, E. Elron; 2N, Parag Winter Pool, 25.iv.2018, E. Elron.

### Key for identification of *Baetis* nymphs in Israel and the Palestinian Authority

**Table:** 

1	Gills with single lamella, claw with single row of teeth, hind wing pads present, carina between eyes absent, setae between mola and prostheca absent in both mandibles, simple prostheca in both mandibles, paraproct without expansion	***Baetis* sensu stricto, 2**
–	At least one of the characters different than above	**other Baetidae**
2	Median caudal filament reduced to 10 segments at most (Figure 4E)	***Baetispacis* sp. n.**
–	Median caudal filament at least 1/2 length of cerci	**3**
3	Gill margins with short spines (Figure 13E). Distal margin of terga with many short spatulas (Figure 13C)	***Baetisnoa* sp. n.**
–	Gill margins without spines (e.g., Figure 6E). Distal margin of terga without spatulas	**4**
4	Gills elongate (almost two terga long). Body elongate and tubular. Terga generally yellow	**5**
–	Gills oval (slightly longer than consecutive tergum). Body somewhat compressed dorso-ventrally. Terga generally brown or with conspicuous brown spots	**6**
5	Triangular spines on distal margin of terga as long as broad (Figure 6C). Maxillary palp not longer than galealacinia (Figure 5F)	***Baetisaureus* sp. n.**
–	Triangular spines on distal margin of terga twice as long as broad (Figure 17C). Maxillary palp longer than galealacinia (Figure 16F)	*** Baetis samochai ***
6	Spines along distal margin of terga regular, triangular (Figure 11C). Coxae without adjacent ventral protuberances. Dorsal margin of femora with blunt setae <1/4 femur width. Claws without subapical setae	*** Baetis monnerati ***
–	Spines along distal margin of terga irregular, blunt, quadrangular (Figure 8C). Coxae with adjacent ventral protuberances (Figure 9). Dorsal margin of femora with blunt setae ~1/3 femur width. Claws with two subapical setae (Figure 8B)	***Baetisgolanensis* sp. n.**

## Concluding remarks

The present work is the first thorough taxonomic study on Israeli mayflies to have been conducted for more than two decades, and increases the Israeli mayfly fauna by 35%. The six *Baetis* species occurring in Israel represent four species groups sensu Müller-Liebenau (1969): the *Baetisrhodani* group (represented by *B.noa*), the *B.lutheri* group (*B.golanensis* and *B.pacis*), the *B.buceratus* group (*B.monnerati*), and the *B.vernus* group (*B.samochai*). *Baetisaureus* may belong in either of the latter two groups. *Baetissamochai* and *B.monnerati* were described from Syria (Koch 1981) and Jordan (Gattolliat et al. 2012), respectively, and the authors predicted that they will also be found in Israel based on their distribution patterns and on comments made in Samocha’s unpublished thesis (1972). Species distribution patterns and the regional hydrography suggest that at least some of the Israeli species are also present in Lebanon and Syria.

Our study was based exclusively on recent collections (2014–2018) by us or by colleagues. Comparison of these newly collected specimens to old material collected by Samocha (early 1970s) and Ortal (1980s–1990s) was impossible due to its poor condition. The collecting details mentioned by Samocha (1972) suggest that our sampling efforts were comprehensive and accurately represent the Israeli fauna. Nevertheless, it is possible that additional *Baetis* species will be found when the old collections are sorted and fully identified. Moreover, our molecular analysis implies that cryptic *Baetis* species are yet to be discovered in Israel.

The highest diversity of *Baetis* species in Israel, as well as for Israeli mayflies in general, is found in the northern stream system (Figure 1), including the upper Jordan River, its tributaries (the Hermon, Senir, Dan, and Iyyon streams) and smaller streams and brooks in the Golan Heights. This pattern can be attributed to the combination of temperate climate, relatively high precipitation, and moderate human disturbance.

Except for the fairly abundant *B.monnerati*, most of the Israeli *Baetis* species face threats that result from the frail status of their habitats. Severe water shortage in recent years affects mainly the northern water courses of Israel, and loss of aquatic habitats due to development or pollution is evident throughout the country. Sensitive *Baetis* populations are likely to degrade accordingly, a trend that has probably already began. Isolated populations of *B.pacis* (Perat Stream) and *B.aureus* (Yarqon Stream) are probably relicts of a continuous distribution pattern in the past, rendering these populations and their habitats of high conservation value.

## Supplementary Material

XML Treatment for Baetis

XML Treatment for Baetis aureus

XML Treatment for Baetis golanensis

XML Treatment for Baetis monnerati

XML Treatment for Baetis (Rhodobaetis) noa

XML Treatment for Baetis pacis

XML Treatment for Baetis samochai

## Supplementary materials

Supplementary material 1 *Baetis* collecting sites with coordinates and altitudes. Data type: list of collecting sites This is an open access article distributed under the terms of the Creative Commons Attribution License (CC BY 4.0), which permits unrestricted use, distribution, and reproduction in any medium, provided the original author and source are credited.Zohar Yanai, Jean-Luc Gattolliat, Netta Dorchin

Supplementary material 2 Collecting details and GenBank accession numbers for the specimens used in the molecular analysis. Codes correspond to those in Figure 3. Data type: list of collecting sites This is an open access article distributed under the terms of the Creative Commons Attribution License (CC BY 4.0), which permits unrestricted use, distribution, and reproduction in any medium, provided the original author and source are credited.Zohar Yanai, Jean-Luc Gattolliat, Netta Dorchin
