# Supplementary material for: Taxonomy of Baetis Leach in Israel (Ephemeroptera, Baetidae)
Source: Zookeys. 2018 Nov 1;(794):45–84. doi: 10.3897/zookeys.794.28214 (PMC6224371; doi:10.3897/zookeys.794.28214)
Supplement: Supplementary material 1 — Baetis collecting sites with coordinates and altitudes. [file zookeys-794-045-s001.pdf]

Supplementary material 1

Taxonomy of *Baetis* Leach in Israel (Ephemeroptera, Baetidae)

Authors: Zohar Yanai, Jean-Luc Gattolliat, Netta Dorchin

Data type: list of collecting sites

Explanation note: *Baetis* collecting sites with coordinates and altitudes.

Copyright notice: This dataset is made available under the Open Database License (http://opendatacommons.org/licenses/odbl/1.0/). The Open Database License (ODbL) is a license agreement intended to allow users to freely share, modify, and use this Dataset while maintaining this same freedom for others, provided that the original source and author(s) are credited.

Link: https://doi.org/10.3897/zookeys.794.28214.suppl1

Locality	longitude	latitude	altitude (m a.s.l.)
Ammud Stream (Poem Spring)	32.9775°N	35.4713°E	540
Ammud Stream (Yaqim Spring)	32.9761°N	35.4659°E	540
Arugot Stream	31.4553°N	35.3805°E	-330
Ayit Stream (Ayit Waterfall)	32.9547°N	35.7539°E	470
Barqan Stream	32.5087°N	35.0225°E	70
Daliyyot Stream	32.8898°N	35.6591°E	-180
Dan Stream (Dafna)	33.2346°N	35.6431°E	160
Dan Stream (Dan)	33.2414°N	35.6505°E	180
Dan Stream (Tel Dan)	33.2473°N	35.6507°E	190
Dawid Stream	31.4687°N	35.3927°E	-350
Divsha Spring	33.0901°N	35.6483°E	150
El-Mahfi Winter Pool	33.1479°N	35.8078°E	1040
El-Muayer Winter Pool	33.0823°N	35.7292°E	750
Enan Springs	33.0837°N	35.5732°E	90
Fit Spring	33.2245°N	35.7052°E	540
Gaaton Junction	33.0155°N	35.1769°E	140
Gamla Stream (Peham Springs)	32.9672°N	35.8201°E	690
Gilbon Stream (downstream Gilbon Spring)	33.0409°N	35.6400°E	80
Gilbon Stream (upstream Devora Waterfall)	33.0445°N	35.6688°E	390
haKefar Spring	32.5713°N	35.1136°E	230
Hermon Stream (Panyas Springs)	33.2483°N	35.6942°E	360
Hula (nature reserve)	33.0751°N	35.6095°E	60
Iyyon Stream (nature reserve)	33.2770°N	35.5846°E	480
Iyyon Stream (Qiryat Shemona)	33.2198°N	35.5961°E	120
Jordan River (Ariq Bridge)	32.9026°N	35.6149°E	-210
Jordan River (Ateret Fortress)	33.0031°N	35.6286°E	60
Jordan River (haHamisha Bridge)	33.0536°N	35.6260°E	60
Jordan River (haPeqaq Bridge)	33.0409°N	35.6294°E	70
Jordan River (Neot Mordekhay)	33.1617°N	35.5931°E	70

Keziv Stream (Hardalit Spring)	33.0430°N	35.1853°E	120
Keziv Stream (Tamir Spring)	33.0415°N	35.2493°E	270
Maymon Spring	33.1124°N	35.6604°E	290
Meron Stream (Meron Spring)	32.9786°N	35.4375°E	700
Orevim Stream	33.1483°N	35.6408°E	70
Parag Winter Pool	32.9583°N	35.8359°E	720
Perat Stream (nature reserve)	31.8348°N	35.3381°E	150
Pezael Springs	32.0509°N	35.4045°E	-30
Qazabiyye Springs	32.9818°N	35.7317°E	440
Rekhesh Stream (Rekhesh Spring)	32.6581°N	35.4728°E	30
Rezaniyya Winter Pool	33.0258°N	35.7540°E	650
Rosh Pinna Stream (Rosh Pinna)	32.9714°N	35.5278°E	540
Samakh Stream	32.8358°N	35.7129°E	-20
Senir Stream (Bet Hillel)	33.1989°N	35.6108°E	80
Senir Stream (nature reserve)	33.2331°N	35.6223°E	130
Tavor Stream	32.6136°N	35.5343°E	-190
Tina Spring	33.0783°N	35.6439°E	-180
Yarqon Stream (national park)	32.1132°N	34.9176°E	20
Yehudiyya Stream	32.8992°N	35.6500°E	-180
Zippori Stream (Ras Ali)	32.7714°N	35.1498°E	50
Zippori Stream (Yivqa Spring)	32.7563°N	35.1747°E	90
Zippori Stream (Zippori Springs)	32.7324°N	35.2731°E	220
