# Supplementary material for: Taxonomy of Baetis Leach in Israel (Ephemeroptera, Baetidae)
Source: Zookeys. 2018 Nov 1;(794):45–84. doi: 10.3897/zookeys.794.28214 (PMC6224371; doi:10.3897/zookeys.794.28214)
Supplement: Supplementary material 2 — Collecting details and GenBank accession numbers for the specimens used in the molecular analysis. Codes correspond to those in Figure 3. [file zookeys-794-045-s002.pdf]

Supplementary material 2

Taxonomy of *Baetis* Leach in Israel (Ephemeroptera, Baetidae)

Authors: Zohar Yanai, Jean-Luc Gattolliat, Netta Dorchin

Data type: list of collecting sites

Explanation note: Collecting details and GenBank accession numbers for the specimens used in the molecular analysis. Codes correspond to those in Figure 3.

Copyright notice: This dataset is made available under the Open Database License (http://opendatacommons.org/licenses/odbl/1.0/). The Open Database License (ODbL) is a license agreement intended to allow users to freely share, modify, and use this Dataset while maintaining this same freedom for others, provided that the original source and author(s) are credited.

Link: https://doi.org/10.3897/zookeys.794.28214.suppl2

Species and codes	Locality	Date	Reference	GenBank accession number
B. monnerati 1	ISRAEL, Jordan River	29.x.2015	Current study	MH827928
B. monnerati 2	PALESTINIAN AUTHORITY, Perat Stream	20.vi.2015	Current study	MH827932
B. monnerati 3	ISRAEL, Tina Spring	10.iii.2017	Current study	MH827926
B. monnerati 4	ISRAEL, Iyyon Stream	05.iv.2016	Current study	MH827923
B. monnerati 5	JORDAN, Wadi Shu'yab	09.xi.2009	Gattolliat et al. 2012	HE651537
B. monnerati 6	ISRAEL, Jordan River	27.v.2014	Current study	MH827927
B. monnerati 7	ISRAEL, Iyyon Stream	05.iv.2016	Current study	MH827922
B. monnerati 8	JORDAN, Wadi Shu'yab	09.xi.2009	Gattolliat et al. 2012	HE651541
B. monnerati 9	ISRAEL, Tavor Stream	04.v.2017	Current study	MH827931
B. monnerati 10	ISRAEL, Yehudiyya Stream	27.iii.2016	Current study	MH827930
B. monnerati 11	ISRAEL, Senir Stream	05.xi.2015	Current study	MH827924
B. monnerati 12	ISRAEL, Enan Stream	17.xi.2016	Current study	MH827925
B. monnerati 13	ISRAEL, Qazabiyye Springs	11.v.2015	Current study	MH827929
B. monnerati 14	JORDAN, Wadi Weida'a	09.xi.2009	Gattolliat et al. 2012	HE651538
B. monnerati 15	ISRAEL, Dawid Stream	07.iii.2017	Current study	MH827933

B. monnerati 16	ISRAEL, Dawid Stream	07.iii.2017	Current study	MH827934
B. monnerati 17	ISRAEL, Arugot Stream	09.v.2016	Current study	MH827935
B. golanensis 1	ISRAEL, Tina Spring	10.iii.2017	Current study	MH827946
B. golanensis 2	ISRAEL, Jordan River	16.x.2016	Current study	MH827947
B. golanensis 3	ISRAEL, Divsha Spring	11.v.2016	Current study	MH827944
B. golanensis 4	ISRAEL, Divsha Spring	11.v.2016	Current study	MH827943
B. golanensis 5	ISRAEL, Jordan River	16.x.2016	Current study	MH827948
B. golanensis 6	ISRAEL, Divsha Spring	06.xi.2015	Current study	MH827945
B. samochai 1	ISRAEL, Hula	29.iv.2015	Current study	MH827952
B. samochai 2	ISRAEL, Gamla Stream	28.iii.2016	Current study	MH827955
B. samochai 3	ISRAEL, Ayit Stream	04.iv.2016	Current study	MH827954
B. samochai 4	ISRAEL, Jordan River	16.v.2016	Current study	MH827953
B. aureus 1	ISRAEL, Gamla Stream	04.iv.2016	Current study	MH827936
B. aureus 2	ISRAEL, Ammud Stream	20.v.2014	Current study	MH827938
B. aureus 3	ISRAEL, Rosh Pinna Stream	15.x.2016	Current study	MH827937
B. aureus 4	ISRAEL, Keziv Stream	07.xi.2015	Current study	MH827939
B. aureus 5	ISRAEL, Gaaton Junction	17.vi.2014	Current study	MH827940
B. aureus 6	ISRAEL, Zippori Stream	19.iv.2016	Current study	MH827941
B. aureus 7	ISRAEL, Yarqon Stream	10.iv.2014	Current study	MH827942
B. noa 1	ISRAEL, Senir Stream	09.iii.2017	Current study	MH827949
B. noa 2	ISRAEL, Maymon Spring	04.iv.2016	Current study	MH827951
B. noa 3	ISRAEL, Maymon Spring	22.vi.2014	Current study	MH827950
B. pacis 1	ISRAEL, Iyyon Stream	05.iv.2016	Current study	MH827956
B. pacis 2	ISRAEL, Senir Stream	17.v.2016	Current study	MH827957
B. pacis 3	PALESTINIAN AUTHORITY, Perat Stream	24.i.2017	Current study	MH827960
B. pacis 4	ISRAEL, Senir Stream	09.iii.2017	Current study	MH827958
B. pacis 5	ISRAEL, Senir Stream	09.iii.2017	Current study	MH827959
Cryptic I 1	ISRAEL, Jordan River	29.x.2015	Current study	MH827962

Cryptic I 2	ISRAEL, Jordan River	11.iii.2017	Current study	MH827965
Cryptic I 3	ISRAEL, Jordan River	16.x.2016	Current study	MH827961
Cryptic I 4	ISRAEL, Jordan River	16.v.2016	Current study	MH827963
Cryptic I 5	ISRAEL, Jordan River	11.iii.2017	Current study	MH827964
Cryptic II 1	ISRAEL, Keziv Stream	07.xi.2015	Current study	MH827966
Cryptic II 2	ISRAEL, Keziv Stream	07.xi.2015	Current study	MH827967
Cryptic II 3	ISRAEL, Zippori Stream	02.iii.2015	Current study	MH827968
Cryptic III	ISRAEL, Dan Stream	17.v.2016	Current study	MH827969
B. lutheri	SWITZERLAND, Luzern, Ettiswil, Rot River	30.iv.2012	Rutschmann et al. in prep.	
B. buceratus	SWITZERLAND, Aargau, Felsenau, Aar River	12.iv.2007	Rutschmann et al. in prep.	
B. vernus	SWITZERLAND, Luzern, Ettiswil, Rot River	30.iv.2012	Rutschmann et al. in prep.	
B. rhodani	SWITZERLAND, Vaud, Ballens	iv.2009	Gattolliat et al. 2015	HG935037.1
